# Utilizing Similarity Measures to Map Chemical Reactivity

**DOI:** 10.1002/anie.202521519

**Published:** 2025-11-20

**Authors:** Robert Rauschen, Dean Thomas, Leroy Cronin

**Affiliations:** ^1^ School of Chemistry University of Glasgow University Avenue Glasgow G12 8QQ UK

**Keywords:** Automation, Cross‐correlation similarity, Digital chemistry, Jaccard index, Reaction Monitoring

## Abstract

The development of fully autonomous chemical synthesis platforms requires robust, real‐time assessment of reactivity that does not rely on prior mechanistic knowledge. Existing methods often depend on predefined reaction models or chemical intuition, limiting their generalizability and adaptability. To address this challenge, we introduce a chemically agnostic approach that quantifies reactivity dynamics by applying similarity metrics to the full informational content of in‐situ spectroscopic data. Using NMR, UV/Vis, IR, and EPR spectroscopy, we demonstrate that spectral similarity trajectories can reliably indicate reaction progress, detect kinetic features such as autocatalysis, and resolve complex behaviours including oscillations. Across diverse reaction classes, this method enabled estimated end‐point detection and kinetic profiling without reaction‐specific tuning. For example, the formation of lophine was followed by NMR at different reaction temperatures revealing Arrhenius‐type kinetics. In a different experiment, the Belousov Zhabotinsky reaction was monitored by UV/Vis and the chemical oscillation with a periodicity of 7.25 s was captured. These results establish a generalizable framework for real‐time, data‐driven reactivity monitoring, representing a critical step toward autonomous synthesis guided by multidimensional spectroscopic feedback.

## Graphical abstract

Autonomous chemistry demands unbiased, adaptable tools for monitoring reactivity. We present a general method using similarity analysis of spectroscopic data, independent of prior knowledge. Demonstrated across diverse techniques and reactions, this approach reveals kinetics and dynamics, establishing a foundation for self‐guided chemical platforms and accelerating the future of automated discovery.

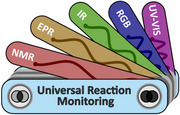

## Introduction

Scientists have long sought to understand the complex processes occurring within a chemical reaction flask. Traditionally, thin‐layer chromatography (TLC) serves a cheap and relatively universal technique to monitor reaction progress and determine when a mixture is ready for further processing.^[^ ^1^, ^2^, ^3^ ^]^ Only then is a comprehensive characterization of the reaction outcome performed after the reaction has concluded. Fundamentally, TLC does not offer deeper structural insights and is limited to *human‐in‐the‐loop* workflows owing to the heavily manual procedure. With advancements in process analytical technology (PAT) however, a growing array of automated techniques allows for real‐time, in situ analysis of reactions, providing critical insights into the formation of intermediates, products and subsequent degradation pathways.^[^ ^4^, ^5^, ^6^, ^7^, ^8^, ^9^, ^10^, ^11^, ^12^, ^13^ ^]^ Due to the complex nature and vast variety of chemical reactions however, chemically‐relevant, automated monitoring is non‐trivial and usually specific to each use case.^[^ ^14^ ^]^ Consequently, the technological solutions to gather reaction data are similarly diverse.^[^ ^15^, ^16^, ^17^, ^18^, ^19^, ^20^ ^]^ With advancing technological capabilities – particularly benchtop analytics – there is a growing repertoire of spectroscopic techniques that afford in situ structural analysis of products formed as well as overall reaction progression. This includes on‐line spectrometers for NMR^[^ ^21^, ^22^, ^23^, ^24^, ^25^ ^]^ IR,^[^ ^22^, ^26^, ^27^ ^]^ UV/Vis,^[^ ^28^, ^29^, ^30^, ^31^, ^32^ ^]^ and EPR spectroscopy^[^ ^33^ ^]^ but also on‐line HPLC^[^ ^34^, ^35^, ^36^ ^]^ and mass spectrometry setups.^[^ ^37^, ^38^, ^39^ ^]^

Despite this impressive suite of ever‐improving technologies, the automated analysis of typically low‐resolution, low‐sensitivity, crude spectra presents a series of challenges. First, not all chemical reactions exhibit diagnostic peaks for tracking reaction progress as signals from reagents, products, and impurities often extensively overlap making quantification case‐dependant.^[^ ^40^ ^]^ Instead of relying on pre‐known regions, one can automatically track the evolution of all distinguishable signals in a spectrum^[^ ^41^ ^]^ however this approach requires powerful peak picking and deconvolution techniques and despite significant spectrum processing development, even machine learning–assisted algorithms still struggle to reliably analyse many datasets, reflecting the high complexity and information density of chemical signals.^[^ ^42^ ^]^ Second, transient species may not yield quantitative signals in viable time frames and/or might not exhibit strong enough responses to external stimuli. Balancing sampling time with signal‐to‐noise is complex as longer analysis times (in terms of spectroscopic scans) may hinder kinetic investigations into rapid reactions.^[^ ^43^ ^]^ Additionally, no single analytical technique is applicable to every chemical problem as some compounds are not UV‐active whilst others do not contain characteristic spin‐active species. Finally, and logistically frustrating, often analytical workflows are accompanied by bespoke, licensed software which can limit interoperability and cross‐compatibility. All of these factors complicate automated analysis, reducing generalised approaches to a case‐by‐case basis.^[^ ^14^ ^]^

**Figure 1:**
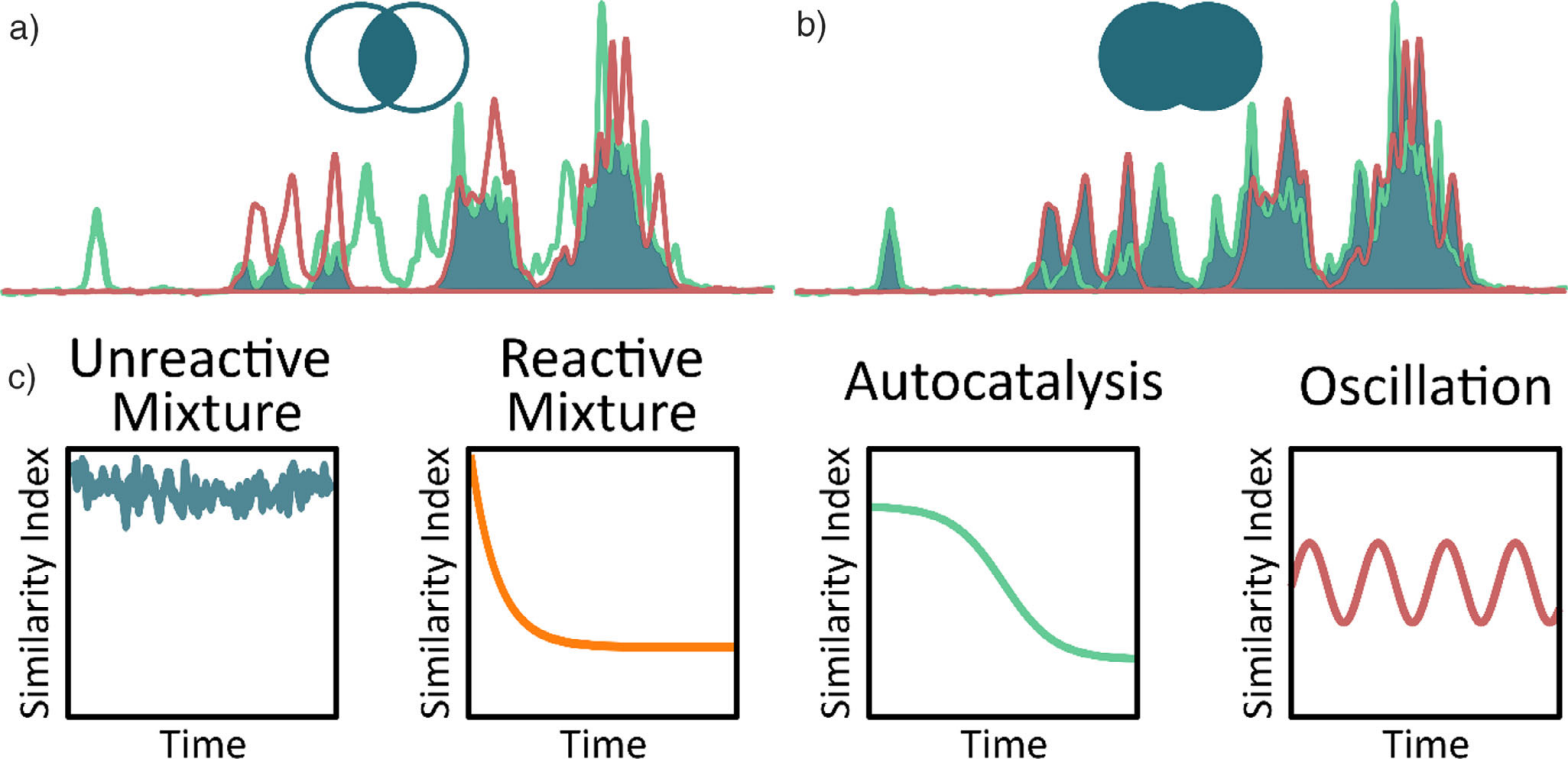
Illustration of the Jaccard similarity index calculation. a) The area under the intersection is divided by b) the area under the union of two spectra to obtain their Jaccard similarity index. c) Exemplar plots of similarity index against time for typical reaction profiles.

An agnostic tool for reaction monitoring is needed that complements multiple spectroscopic techniques to simplify and harmonise the analysis of data for rapid fingerprinting.^[^ ^22^ ^]^ Herein, we propose that similarity metrics like cross correlation or intersection over union (Jaccard index),^[^ ^44^, ^45^ ^]^ which are widely used in statistics for comparing the similarity of series and sample sets, respectively, can serve as a proxy for extracting comprehensive information about reactivity from spectroscopic data (Figure 1). In the context of spectral analysis, we adopt a geometric interpretation of the similarity index as follows: the intersection of two spectra can be calculated by choosing the minimum intensity data point at each chemical shift/wavelength and integrating the resulting curve (Figure 1a). The union is derived from the maximum intensity, accordingly (Figure 1b). Instead of disentangling the peaks in a spectrum, obtaining a similarity metric is sufficient to draw information about the reaction progression by comparing how “dissimilar” the spectra become over time in comparison to the first spectrum in the sample set. At the beginning of the experiment, the first spectrum is compared to itself, giving a Jaccard index with a maximum value of 1.0. Thereafter, it varies as a function of the reaction profile (Figure 1c).

The Jaccard index itself is then obtained by dividing the absolute value of the intersection area (|*A* ∩ *B*|) by the union area (|*A* ∪ *B*|) (Equation 1).

(1) $$J\left(A,B\right)=\frac{\left|A\cap B\right|}{\left|A\cup B\right|}$$

When it comes to the comparison of two time‐dependant data series ***f*** (***t***) and ***g*** (***t***) where the geometrical integration is not chemically plausible, we use the maximum cross‐correlation and normalise it with the mean autocorrelation to measure the similarity ***S*** (***f***, ***g***) (Equation 2).

(2) $$S\left(f,g\right)=\frac{\max_{\tau \varepsilon \left[0,t\right]}\left(f*g\right)\left(\tau \right)}{\sqrt{\sum_{\tau =0}^{t}f{\left(\tau \right)}^{2}\;\cdot \;\sum_{\tau =0}^{t}g{\left(\tau \right)}^{2}}}$$

Of which, the cross‐correlation is a function of the shift ***τ*** (Equation 3) where $\bar{f(t)}$ denotes the complex conjugate of ***f*** (***t***).

(3) f∗gτΔ=∑t=−∞∞ft¯·gt+τ

This cross‐correlation similarity is particularly useful when comparing raw free induction decay (FID) data from NMR experiments (vide infra Figure 6c) as well as in cases where analysis with the Jaccard index is inappropriate as negative areas would lead to cancellation effects.

Assuming that the initial state of a reactive mixture is known, i.e., a spectrum was acquired before the reaction started, the similarity between the initial spectrum and the spectrum at a given timepoint can serve as a proxy for the reactivity of that mixture. This is particularly valuable for automated platforms requiring autonomous decision‐making capabilities as traditionally, case‐specific reaction monitoring required either predefined characteristic properties or *human‐in‐the‐loop* intervention during execution.

As many chemical transformations are performed in the solution phase, a solution‐based automation platform equipped with a wide array of in situ and on‐line monitoring technologies was required to ensure real‐time feedback and high reproducibility.^[^ ^14^ ^]^ The modular Chemputer^[^ ^46^, ^47^, ^48^ ^]^ platform served as the operational backbone owing to the flexible liquid‐handling architecture alongside the broad suite of integrated sensing modalities,^[^ ^49^ ^]^ making it ideally suited for method development across diverse chemistries. Crucially, the Chemputer supports both standard and user‐defined experimental workflows, enabling direct integration of spectroscopic inputs for intelligent reaction control.^[^ ^31^ ^]^ These characteristics are particularly well‐suited for evaluating analysis strategies aimed at general applicability, where minimal assumptions are made about the underlying reaction mechanisms or reagent identities.

Beyond liquid handling and analysis, frameworks such as the Chemputer embody the foundational principles of digital chemistry,^[^ ^50^ ^]^ i.e., capturing all procedural, physical, and analytical steps as codified instructions. This formalization enables automated error detection and correction through reaction feedback. For instance, deviations in real‐time analytical signals, such as stalled reactivity or unexpected side‐product formation, can trigger workflow modifications, such as reagent re‐dosing or extended reaction times,^[^ ^51^ ^]^ thereby closing the loop between observation and execution. This approach to feedback‐driven adaptivity is essential for achieving truly autonomous chemical synthesis.

Importantly, benchtop NMR, IR, UV/Vis, and EPR spectrometers have become increasingly viable for integration into automated synthesis platforms due to their reduced footprint, cost, and user‐friendly design. These instruments now offer sufficient sensitivity and resolution for reaction monitoring in many cases ^[^ ^52^ ^]^ and require only minor hardware modifications (e.g., flow cell attachments) and software extensions to interface with existing infrastructure.^[^ ^31^ ^]^ Their compatibility with open‐source control frameworks and standard data communication protocols makes them ideal candidates for closed‐loop automation, where spectroscopic feedback can be used not just for data logging but for active decision‐making. (For detailed implementation strategies, including hardware schematics and software integration pathways, see Supporting Information, Sections 1.2 and 2).

## Results and Discussion

**Figure 2:**
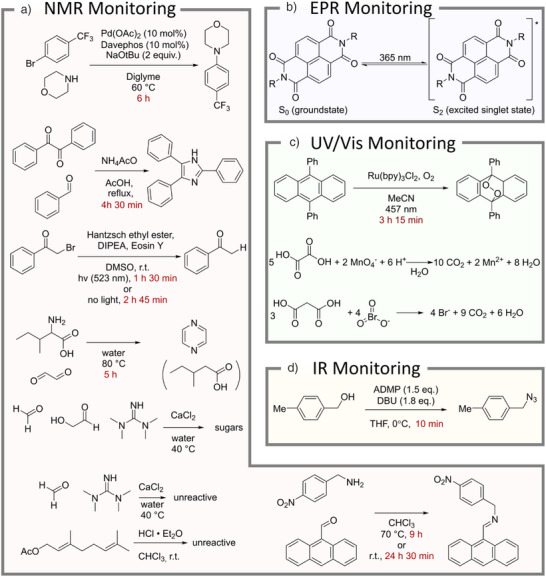
Overview of Reactions Subjected to Reaction Monitoring. Where applicable, reaction times in red refer to the time after which the similarity index of the spectra reached a plateau. Unreactive, oscillating, and autocatalytic mixtures were purposefully included in this scope to demonstrate how the similarity‐based monitoring approach can distinguish different kinetic behaviours.

To investigate the versatility of this reaction monitoring approach, the algorithm was applied to a range of different chemistries and spectroscopic techniques (Figure 2). A variety of different chemical reactions including imine formations,^[^ ^51^ ^]^ formose reactions,^[^ ^53^ ^]^ Buchwald‐Hartwig aminations,^[^ ^54^ ^]^ Maillard reactions,^[^ ^55^, ^56^, ^57^, ^58^, ^59^, ^60^ ^]^ heterocycle formation,^[^ ^61^, ^62^, ^63^, ^64^, ^65^ ^]^ photocatalytic debromination,^[^ ^66^ ^]^ and others were executed on the Chemputer platform and monitored using the on‐line sampling sequences. The scope of reactions included those with known and unknown outcomes to examine the performance of the monitoring algorithm with minimal bias in chemical space. All reactions were subjected to flow‐cell sampling in regular intervals, depending on the number of scans acquired. When the plateau detection indicated that the reaction had finished, the reactions were quenched, the product phase was isolated via liquid‐liquid extraction and purified when a single product was expected or subjected to chromatographic analysis if multiple undefined products were expected.

**Figure 3:**
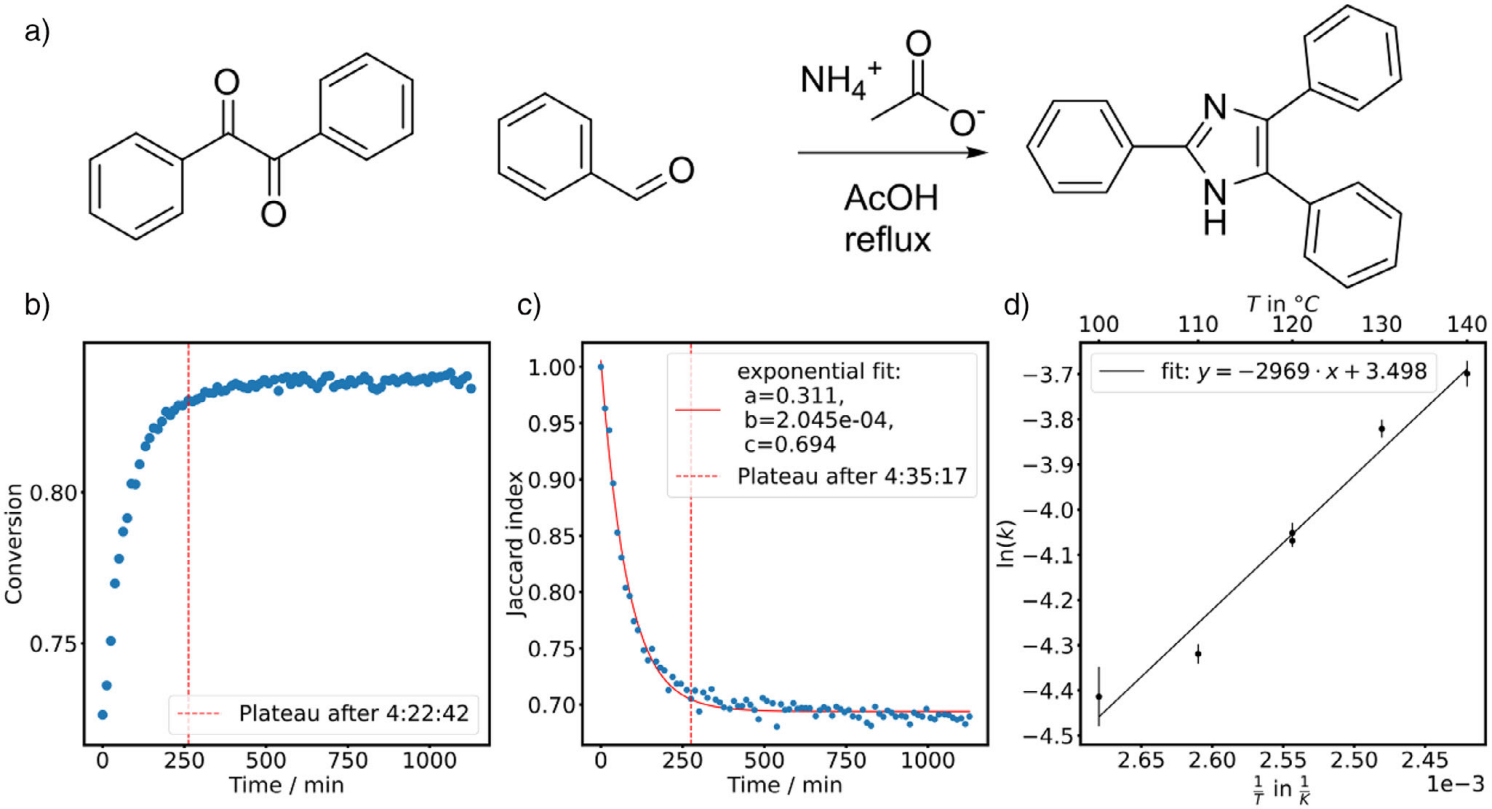
Exemplar monitoring data from Lophine formation. a) Reaction scheme. b) Tracking of the conversion over time by comparing diagnostic NMR signals of the starting material and the product in the aromatic region against each other. c) Similarity index of the NMR spectra over time compared against the initial spectrum following an exponential decay that reaches a plateau around the same time as the conversion time series. d) Arrhenius analysis of the Jaccard index at different temperatures: The time series of the Jaccard index can be fitted well with an exponential decay function and the time decay constant from that fit function was plotted against the reaction temperature. Data obtained with an 80 MHz bench‐top NMR spectrometer.

Considering NMR first, whenever possible, a solvent suppression pulse sequence was used to allow for the accurate tracking of reagents in non‐deuterated solvents. Preliminary test acquisitions were concluded in advance to optimise the suppression parameters via Bayesian optimisation (see Figure S1). When diagnostic signals for the product and the starting material were available, their integrals were measured, resulting in a benchmark conversion for comparison over the course of the reaction (see Figure S3 for an example of conversion time series). Satisfyingly, for the synthesis of lophine (Figure 3a), the time point at which the Jaccard plot reaches a stable plateau (Figure 3c) compares well with the plateau time index obtained from a traditional conversion plot derived from analysing diagnostic signals only (Figure 3b). The plateau was defined to be stable when the slope from a linear regression of five consecutive data points falls below a threshold value of 1 × 10^−3^.

To demonstrate that the similarity of a sequence of spectra provides a robust measure of the reaction progress and is not instead analysing the evolution of noise or the shift of instrument parameters, the reaction kinetics of lophine's formation was investigated at different temperatures. To this end it has been recently demonstrated^[^ ^67^ ^]^ how signal averaging during post‐processing can be exploited to simultaneously obtain adequate signal‐to‐noise ratio and temporal resolution which we extensively utilised whenever temporal resolution is critical. The reaction vessel was held at a constant temperature and aliquots from the reaction were analysed with NMR at regular intervals. An exponential decay function was fitted to the course of the similarity index to extract a time constant, which was subsequently plotted against the reaction temperature. As can be seen (Figure 3d), a linear relationship emerges, which indicates that the performed analysis is a good measure for the physical kinetic properties of the reaction.

Having established that a plateau in the similarity metric is insightful for reaction endpoint detection, the next logical step is to understand how the Jaccard index categorises unreactive or unchanging systems. When monitoring a blank sample, containing just solvent, the Jaccard index fluctuated randomly leading to erratic behaviour of the plateau‐detection algorithm. To avoid this, noisy datasets can be detected by calculating their Spearman correlation coefficient (see Supporting Information, Section 5) and excluded from monitoring. Although this serves as a practical workaround it does not explain the erratic behaviour of the Jaccard index when the underlying spectra are very similar. To understand this, a deeper mathematical investigation of the Jaccard algorithm was carried out (see Supporting Information, Section 6). The numerical analysis revealed that the application of the minimum (or maximum) function on the intensity values in the experimental spectrum is mathematically “ill‐conditioned” meaning that it can arbitrarily amplify the experimental error of the intensity values in certain cases. This phenomenon is known in the context of subtraction in floating point arithmetic where it leads to so‐called “catastrophic cancellation”.^[^ ^68^ ^]^ Having identified the ill‐conditioned minimum function as the problematic part of the algorithm, a mitigation strategy is clear. Prior to the calculation of the Jaccard similarity index, the instrument error on the intensity values is estimated and whenever the difference between two intensity values during the comparison of two spectra is smaller than the instrument error, the values are treated as equal. This modified procedure reliably avoids catastrophic error amplification and correctly identifies unchanging reactions.

The similarity score was then validated over a diverse set of chemical reactions (see Supporting Information, Section 3), demonstrating that the agreement between the plateau time of the conversion plot and the Jaccard plot is reproducible. Similar plateau‐detection approaches with alternate reaction properties could also have been taken, e.g., heat flux for exothermic/endothermic reactions, pressure change for reactions which evolve gas, or colour change if UV/Vis‐active compounds were part of the reaction (See Supporting Information, Section 3.8 for the photochemical oxidation of an anthracene derivative). It should be noted however that plateau‐detection approaches are not suitable for transient intermediates or the characterisation of oscillatory behaviour. Only systems that reach a long‐term stable state are amenable to endpoint detection analysis.

Inspired by the robust results in NMR, the similarity‐based analysis approach was applied to spectra obtained with electron paramagnetic resonance (EPR) spectroscopy as well. As the paramagnetic nature of species with unpaired electrons interferes with the signal obtained in NMR experiments, EPR spectroscopy serves as a complementary analytical technique, often providing critical insights into transition‐metal containing complexes, thereby opening another fraction of chemical space to automated reaction monitoring. The detected signal in an EPR experiment is normally proportional to the first derivative of the absorption due to the lock‐in detection method,^[^ ^69^ ^]^ which improves sensitivity and noise suppression. Consequently, the data was integrated as part of the pre‐processing before applying the similarity measures to obtain a traditional absorption‐mode spectrum. Initially, the time‐resolved monitoring of the photochemical excitation of a naphthalene diamide (NDI) gel was chosen to explore the applicability of the Jaccard similarity index to integrated EPR data. Naturally, the first spectrum in the dataset will appear as a flat baseline because no EPR signal is expected at the beginning of the excitation period. This means the similarity index will rapidly decay to zero when taking the first spectrum as a reference (see Supporting Information, Section 3.11), which holds limited information in this case. In fact, it is more insightful to compare against the last spectrum in the dataset to find out if the excitation process reached an equilibrium. The resulting Jaccard indices clearly show that the excitation process has not reached an equilibrium within the 50 min of monitoring.

**Figure 4:**
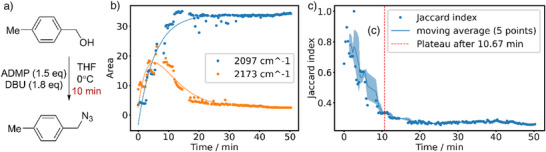
IR Monitoring of Azide Formation. a) Reaction scheme. b) Isolated, diagnostic peaks can be analysed for kinetic information about intermediates. c) The overall Jaccard index suffices to detect when the reaction is mostly completed.

Next, the same approach was demonstrated with on‐line IR spectroscopy (Figure 4). IR spectra are infamously hard to deconvolute, especially in the fingerprint region because of the vast number of overlapping signals and the richness in chemical information. This makes IR spectra particularly interesting for a meta‐analysis with similarity metrics. The in‐situ formation of azides from alcohols was monitored over a total period of 50 minutes in a spectral window between 1500 cm^−1^ and 2500 cm^−1^ and it was found that there are two peaks in the spectrum that are amenable to isolated analysis. The product azide peak at 2097 cm^−1^ grows over time, following an exponential trend that is characteristic for first‐order kinetics. Moreover, there is a transient intermediate, observed at 2173 cm^−1^ which follows an exponential “rise‐and‐fall” curve.^[^ ^70^, ^71^ ^]^ Analysing the similarity of the whole spectrum over time leads to the conclusion that the reaction nears completion after roughly 10 minutes.

Overall, the reactions where the similarity index reaches a stable plateau were found to follow apparent first‐order kinetics, meaning that monitoring it through spectroscopic techniques will most likely yield a data series that can be fitted with a simple exponential function. The similarity‐based analysis is not restricted to these cases though. This approach is also valuable for complex systems, where dynamic behaviours such as switching and oscillations are known to propagate through complex networks to drive material change.

**Figure 5:**
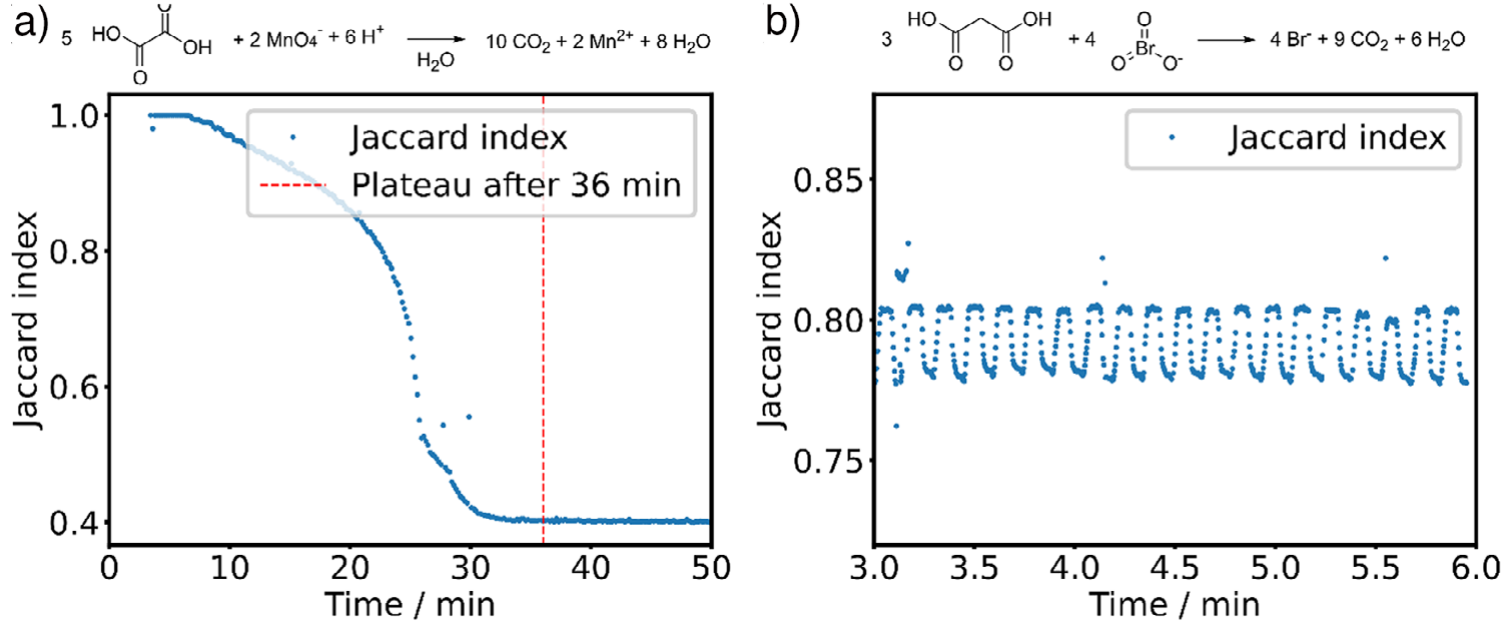
Complex kinetic behaviour like oscillation and autocatalysis. Similarity metrics can also be used to track reactions that have kinetic profiles more complex than apparent first‐order exponential time series. a) The autocatalytic behaviour of the permanganate‐oxalate‐reaction is easily detected by the sigmoidal shape of the time series. b) The chemical oscillation frequency of the Belousov‐Zhabotinsky reaction can be obtained by Fourier transformation of the time series of the similarity index of the UV/vis spectra.

A model example for non‐standard kinetics is the reaction between potassium permanganate and oxalic acid,^[^ ^72^ ^]^ owing to the autocatalytic behaviour as soon as critical amounts of Mn(II) are present in solution. A variety of kinetic models^[^ ^72^, ^73^, ^74^ ^]^ have been developed to describe the kinetics of this reaction and a numerical simulation of the time course of the concentration of relevant species was carried out based on a recent kinetic model to find out that the species with the most prominent UV/Vis signal (permanganate) is expected to follow a sigmoidal decay function (see Supporting Information, Section 3.9). Monitoring in short intervals and similarity‐based analysis of the resulting spectra confirmed that the data is, indeed, following a sigmoidal function (Figure 5a).

Next, a reaction was chosen that does not reach a stable plateau initially because it displays oscillatory behaviour. The Belousov‐Zhabotinsky reaction is the most famous example of such oscillatory behaviour and can be readily monitoring through UV/Vis spectroscopy (Figure 5b). The analysis of the similarity of the resulting spectra revealed the chemical oscillation with a period of 7.25 s (See Figure S10).

**Figure 6:**
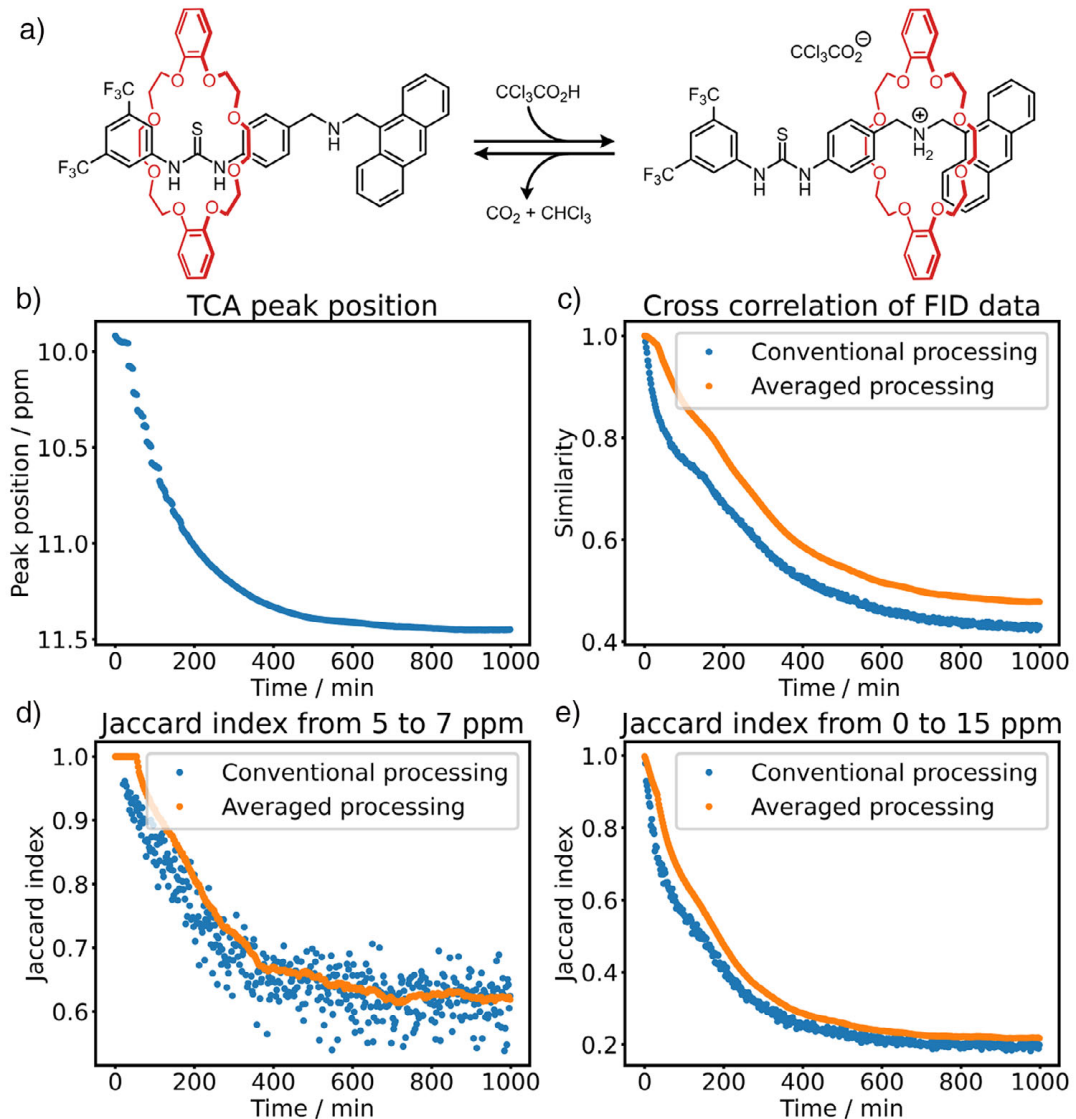
Molecular Switching Insights. a) The programmable shuttling of the macrocycle between binding sites of a [2]rotaxane can be monitored using various analytical approaches, selected according to the specific experimental objective and the level of prior knowledge available. For instance: b) Tracking the CCl_3_CO_2_ *H* (TCA) chemical shift, which is sensitive to pH, provides a direct readout of fuel decomposition over time. c) The averaged free induction decay (FID) similarity over time offers a global, unsupervised view of spectral evolution. d) The Jaccard index calculated within the 5–7 ppm region highlights local spectral changes associated with protonation‐state‐dependent switching. e) The Jaccard index computed across the full spectrum captures overall compositional or dynamic change throughout the switching cycle.

Finally, functional assemblies can undergo controlled switching between distinct states in response to chemical fuels or environmental stimuli. NMR spectroscopy was employed to investigate the switching of a [2]rotaxane using trichloroacetic acid (TCA), providing an automated platform for observing the response over time (Figure 6). The macrocycle shuttles between two distinct positions depending on the protonation state of the system. A single pulse of trichloroacetic acid (CCl_3_CO_2_H), transiently protonates the rotaxane and induces shuttling before undergoing base‐catalysed decarboxylation and decomposing into carbon dioxide (CO_2_) and chloroform (CHCl_3_) as the only waste products. During this process, basic conditions in the medium are restored, reversing the macrocycle's position and completing one full operational cycle (Figure 6a). Owing to the dynamic strengths of the similarity approach, the cycle can be monitored along multiple reaction coordinates – ranging from chemical conversion, as seen in the changing CCl_3_CO_2_ *H* chemical shift values (Figure 6b), to spectral pattern evolution measured by local (5–7 ppm) or global Jaccard index values (Figure 6d,e, respectively), as well as overall signal similarity through time‐resolved FID comparison (Figure 6c). Each approach offers distinct insights into the [2]rotaxane's operation and can be tuned depending on the analytical focus. Such systems are ideally suited for optimisation with autonomous workflows, which can iteratively adjust reaction conditions in response to feedback, accelerating the development of molecular machines capable of performing complex, adaptive functions.

## Conclusion

Whether utilising bespoke and expensive analytical platforms or simpler, commercially available sensors, the pressing need to analyse and understand reactions is ubiquitous across academic and industrial sciences. This workflow however relies heavily on the skills and intuition of trained experts, which is challenging to fully capture in autonomous workflows. To address this, we have developed a simple yet powerful, chemically agnostic algorithm that analyses of the similarity of spectroscopic data over time. The broad applicability of this approach was demonstrated across the fundamental pillars of analytical spectroscopy (NMR, EPR, IR and UV/Vis) to gain a plethora of insights into reactivity for various chemical scenarios. Different kinetic behaviours, including apparent first‐order kinetics, oscillations, and autocatalysis are identified in the analysis of the spectroscopic similarity, depending on the spectroscopic technique and the obtained signal type. Furthermore, the series of similarity values can be subjected to a plateau‐detection algorithm to determine the endpoint of reactions. This information can then be used to autonomously trigger more complex sub‐routines including quenching or work‐up procedures upon reaction completion. The applications of this approach open the door to fully autonomous reaction monitoring and control, enabling real‐time decision making in chemical synthesis without the need for prior mechanistic knowledge or expert intervention.

## Supporting Information

The python notebooks to reproduce the figures presented throughout the manuscript and the supplementary information, along with the entire dataset, were uploaded to Zenodo.org, doi: 10.5281/zenodo.17241207. The necessary software for reproducing the plots can be obtained from GitHub: github.com/croningp/spectroscopic_similarity. The software framework for operating the Chemputer platform is subject to change and the active development version of the software can be made available to collaborators upon request to the corresponding author. A referenced version of the software stack^[^ ^49^ ^]^ is publicly available on Zenodo, doi: 10.5281/zenodo.6534009.

## Author Contributions

**R.R**.: Conceptualization, Data curation, Formal analysis, Investigation, Methodology, Project administration, Software, Validation, Visualization, Writing — original draft, Writing — review & editing. **D.T**.: Supervision, Visualization, Writing — review & editing. **L.C**.: Conceptualization, Funding Acquisition, Resources, Supervision, Writing — review & editing

## Conflict of Interests

The authors declare no conflict of interest.

## Supporting information



Supporting Information

## Data Availability

The data that support the findings of this study are openly available in zenodo at [10.5281/zenodo.1724120], reference number 17241207.
