# Supplementary material for: Utilizing Similarity Measures to Map Chemical Reactivity
Source: Angew Chem Int Ed Engl. 2025 Nov 20;65(2):e21519. doi: 10.1002/anie.202521519 (PMC12790357; doi:10.1002/anie.202521519)
Supplement: Supplementary file 1 — Supporting Information [file ANIE-65-e21519-s001.pdf]

SUPPORTING INFORMATION

Utilizing Similarity Measures to Map Chemical Reactivity

Robert Rauschen[1], Dean Thomas[1] & Leroy Cronin[1*]

[1]*Advanced Research Centre, University of Glasgow, 11 Chapel Lane, Glasgow, G11 6EW, UK.*

*Corresponding author(s). E-mail(s): lee.cronin@glasgow.ac.uk;

Contents

1. Materials and Instrumentation ... 4
1.1 Materials ... 4
1.2 Chemputer platform ... 4
1.3 Offline NMR measurements ... 5
1.4 Electron paramagnetic resonance (EPR) measurements ... 5
1.5 General procedure for online UV/Vis measurements ... 5
1.6 General procedure for on-line NMR measurements ... 6
2 Custom XDL steps ... 9
2.1 XDL step for data acquisition ... 9
2.2 Reaction monitoring using similarity measures ... 10
2.2.1 Slope detection within the *Monitor* step ... 11
2.2.2 Calculation of the Jaccard similarity index ... 13
3 Experimental procedures ... 15
3.1 Acyclic Terpene precursor under acidic conditions ... 16
3.2 Formose reaction ... 17
3.3 Maillard reaction ... 19
3.4 Imine formation ... 20
3.5 Photochemical debromination ... 22
3.6 Formation of Lophine ... 24
3.7 Buchwald-Hartwig amination ... 25
3.8 Photochemical oxidation of 9,10-Diphenylanthracene ... 27

3.9 The reaction between potassium permanganate and oxalic acid 29

3.10 Belousov-Zhabotinsky reaction 30

3.11 Photochemical excitation of a naphthalene diamide (NDI) gel 32

3.12 Azide formation 34

3.13 Molecular switching of a rotaxane 35

4 Reaction monitoring *via* conversion tracking 36

5 Spearman correlation coefficient to detect noisy data 39

6 Numerical analysis of the Jaccard algorithm 41

6.1 Effect of the error correction upon calculation of the Jaccard index 44

7 Comparison of different directionalities when calculating the similarity 46

8 Calculations for kinetic analysis 47

9 References 48

1. Materials and Instrumentation

1.1 Materials

The reagent-grade chemicals were obtained from Fluorochem, Sigma-Aldrich and TCI. All reagents were used as obtained without further purification. Solvents were purchased from several departmental suppliers, Honeywell, Fisher, and Sigma-Aldrich.

1.2 Chemputer platform

The pumps, valves, and frames of the Chemputer backbone are standardised pieces of equipment designed by the Cronin Group and were assembled as described in previous publications. Fittings, adaptors, tubing, and other commercially standardised parts are purchased from respective suppliers. The exact part numbers for a standardised platform alongside extensive build instructions are readily available. [1]

Building on this work, the software representation of the flow cell setup that is used to sample reactions *via* the Chemputer backbone was refined. The refinement included treating the flow cell as a 'cartridge'-type node, which means that liquids can be passed through that node, but it cannot act as a sink or source for a liquid transfer. This definition in the software ensures that there are always two pumps involved in a flow cell transfer: one pump is pushing liquid into the flow cell from the bottom and the other pump is pulling out the same volume at the top of the cell. This setup ensures that there is no pressure building up in the system even though it is completely sealed and, thereby, protected from the ambient atmosphere. To empty the cell, the flow direction is inverted, and air or inert gas is used to purge the cell from the top. It should be noted at this point that the liquid movement is stopped during the spectrum acquisition to avoid perturbations. In more traditional terms, this setup is usually referred to as stopped-flow mode.

A detailed explanation of the flow cell setup with the benchtop spectrometers can be found in the Chemputer manual from the Cronin group.[1]

1.3 Offline NMR measurements

NMR measurements were performed with Bruker Avance III HD 600 spectrometer operating at 600.1 and 150.9 MHz for ^{1}H and ^{13}C, respectively. Spectra were collected at 298 K, chemical shifts are reported in ppm and were calibrated for the (residual) NMR solvent signal (multiplicities are given as s: singlet, d: doublet, t: triplet, q: quartet, m: multiplet, with coupling constants reported in Hz). The spectra were processed with MestReNova 14.0.0.

1.4 Electron paramagnetic resonance (EPR) measurements

All EPR data were recorded at X-band frequency (9.67 GHz) on a Bruker ELEXSYS E500 spectrometer equipped with an ER 4102ST-O optical transmission resonator. All measurements were collected using 10 mg/mL buffered aqueous solutions. Solutions were transferred into soda glass capillary tubes with a 2 mm diameter. Capillary tubes were sealed at one end and samples filled 20 mm of the tube. EPR measurements were performed by Dr Stephen Sproules.

1.5 General procedure for online UV/Vis measurements

UV/Vis spectra were acquired with a DH-2000 light source and a FIA-Z-SMA 905 PEEK (10 mm path length) flow cell from Ocean Optics, connected by fibre optics to an Avantes AvaSpec-DUAL 4096 spectrophotometer. The light source was manually switched on prior to execution of experiments employing UV/Vis spectrophotometry and manually turned off afterwards. The reference spectra for required solvents were acquired manually just before starting experiments on given day and used for further processing of the raw data.

[1] https://croningroup.gitlab.io/chemputer/manual-automation/hardware/spectrometer/spinsolve_nmr.html

1.6 General procedure for on-line NMR measurements

NMR measurements were performed with the following benchtop Magritek Spinsolve spectrometers.

- Spinsolve 80 Ultra Multi-X SPA3681
  - *Nuclei*: ^{1}H (80 MHz), ^{19}F (76 MHz), ^{11}B (26 MHz), ^{31}P (32 MHz)
- Spinsolve 80 Ultra Carbon SPA3422
  - *Nuclei*: ^{1}H (80 MHz), ^{19}F (76 MHz), ^{13}C (20 MHz)
- Spinsolve 60 Ultra Carbon SPA1111
  - *Nuclei*: ^{1}H (60 MHz), ^{19}F (58 MHz), ^{13}C (15 MHz)

Spectra were collected at 298 K and chemical shifts are reported in ppm. Because most reactions use solvents which are not readily (or cheaply) available in deuterated form, the spectra were recorded in non-deuterated solvent by default and referenced with respect to the strongest solvent signal. For approximation, the following chemical shifts were assumed:

- Dichloromethane: 5.30 ppm
- Chloroform: 7.26 ppm
- Acetonitrile: 2.0 ppm
- Acetic acid: 1.8 ppm
- Water: 4.79 ppm
- Diglyme: 3.20 ppm
- DMSO: 2.50 ppm

Furthermore, to allow the analysis of reagents in low concentration, the solvent suppression mode of the Spinsolve spectrometers was applied extensively. The solvent suppression mode comes with a range of custom parameters to fine-tune the suppression:

- a correction factor to adjust the suppression power.

- the number of dummy scans (used to automatically determine the frequency of the strongest signal to be suppressed)
- the acquisition time.
- the repetition time.

The latter two should be adjusted to match the spin-lattice relaxation time of the NMR experiment (commonly denoted as T_1). To find a good set of parameters for suppression experiments in a time-efficient manner, a calibration sample of 1,4-bis(trimethylsilyl)benzene in non-deuterated chloroform was prepared and the ratio between the integrals of the methyl signal of the compound and the solvent signal was defined as the objective for optimisation. A Bayesian algorithm [2] was then used to generate a set of parameters to conduct an NMR experiment, the result of which was used to update the optimiser before the next set of parameters was generated. The nature of the Bayesian optimisation algorithm will not be explained in detail here because extensive reviews have been written about Bayesian optimisation before [3,4]. The algorithm converged quickly (after less than ten iterations) to a maximum value for the ratio of analyte to solvent around a correction factor of 1.04 (Figure S1). The objective is plotted with a negative sign due to the internal mechanics of the optimisation algorithm. The other parameters did not seem to affect the integrals substantially; this could have been expected because the number of scans and the measurement time should merely influence the signal-to-noise ratio and not the integrals under the signals.

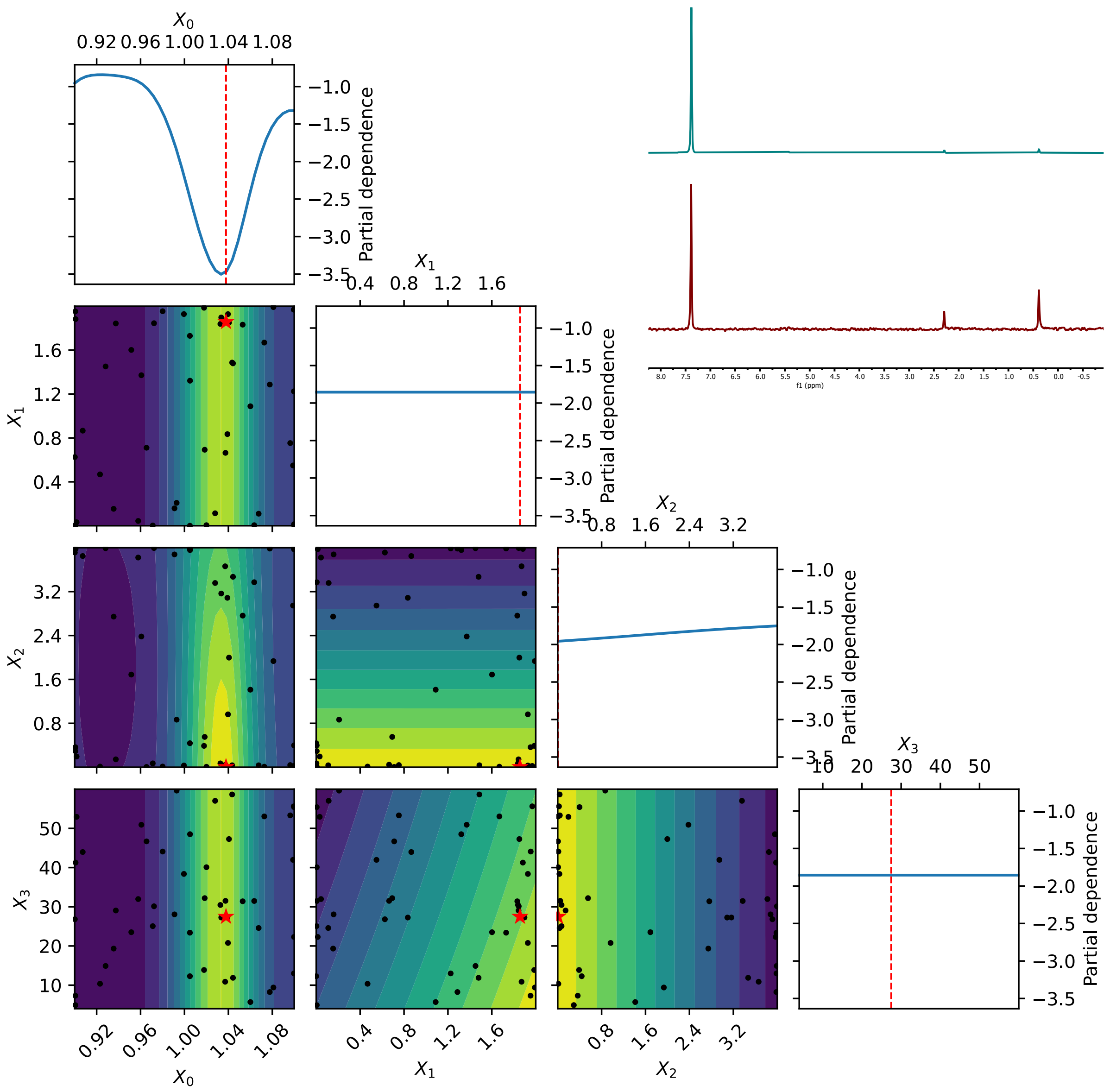

Figure S1: Results for optimising the ratio between analyte and solvent signal in a solvent suppressed NMR spectrum on the Spinsolve bench-top spectrometer. Bayesian optimisation was applied to find an ideal combination of suppression parameters. X_0: correction factor, X_1: number of dummy scans to find the suppression frequency, X_2: acquisition time, X_3: repetition time. The inset in the top right corner shows how the suppression successfully reduces the intensity of the solvent signal relative to the analyte signals.

The spectra were processed with in-house python software.[2]

Next, the sampling sequence was improved because it was found that the quality of the produced spectra would decay rapidly if the machine was not shimmed regularly. The impaired quality of the spectra mostly manifests in an accumulating horizontal shift of the spectrum due to the internal drift of the instrument's magnet. While small shifts can be corrected by referencing the spectrum to a signal with known chemical shift, the accumulated drift would at some point cause the hardware lock to fail, leading to a corrupted acquisition. To mitigate this, regular shims need to be conducted. A reference shim on a calibration sample before the experiment followed by a quick shim on the solvent signal in the sample every 10th measurement was shown to be sufficient for delivering reliable data in most cases.

2 Custom XDL steps

The platform (a standard implementation of Chemputer as described by Rohrbach et al. [1]) was operated using XDL v2.3.1a8 (development version[3] as of July 2024). The additional XDL steps to run the analytical extensions in form of benchtop NMR and UV/Vis spectrometers were adopted from previous projects [5] and updated as follows.

2.1 XDL step for data acquisition

An abstract ***FlowCellSampling*** was developed to cover the sampling procedure for different kinds of analytical devices in a single step. The ***FlowCellSampling*** step takes care of priming the flow cell before conducting the measurement and is also responsible for returning the aliquot to the appropriate reactor (or disposing of it if required) after the spectrum acquisition is completed. Upon sampling the step will automatically invoke an analytical XDL base step to acquire the correct kind of spectrum depending on the node type of the analytical node that was

[2] https://gitlab.com/croningroup/chemputer/analyticallabware
[3] https://gitlab.com/croningroup/chemputer/chempu-project

specified as a step parameter. In the current implementation, two spectroscopic techniques are fully supported by the XDL software stack with the ***RunNMR*** and the ***RunUV*** steps.

2.2 Reaction monitoring using similarity measures

The standard XDL implementation comes with a mechanism for monitoring individual values in a loop. Each time the ***Monitor*** step is executed, it acquires the most recent reading and compares it against a minimum and a maximum threshold value to find out if the execution is to be continued or aborted. To efficiently exploit the capabilities of spectroscopic monitoring, two additional features were added to this implementation: first, a wrapper was added to the ***Monitor*** step to allow the comparison of the current derivative of the data sequence instead of the absolute magnitude of the data values. This allows a more flexible design of input files for experiments where the magnitude of the values that are to be monitored is unclear at the beginning of the experiment. A preliminary implementation of this mechanism was already described previously. [5]

2.2.1 Slope detection within the *Monitor* step

The calculation of the derivative has several caveats to account for problematic or noisy data. First, the responsible function will remove any invalid NaN values from the list of data points to avoid errors in the linear regression later. It will also check if there are at least as much data points available as requested by the user input. Furthermore, the Spearman correlation index is calculated to avoid erroneous slope calculations where the data is too noisy to represent a smooth curve. The threshold value of 0.5 for the Spearman correlation coefficient was arbitrarily chosen based on experimental experience. Finally, if all checks pass, the datapoints are normalised against the maximum data point and their slope is calculated using linear regression.

```
def record_slope(self, readings: list[float] | np.ndarray):
        """Records the slope of the last n sensor readings.
        First, the function removes invalid values like Nones and NaNs
        and checks if enough datapoints have been acquired.
        Then, it checks if the data is too noisy to detect a slope by calculating the
        spearman correlation coefficient. If the data is too noisy, the function aborts.
        If the data is not too noisy, the function calculates the slope of the last n
        datapoints via linear regression and stores the absolute value of the slope."""
        # remove nan values from evaluation
        no_nan_readings = [
            num for num in readings if num is not None and not np.isnan(float(num))
        ]
        if self.slope_valid is False:
            self.logger.info("Too few datapoints acquired, entering next cycle...")
            self.current_reading = None
            return
        # check if enough datapoints have been acquired
        if len(no_nan_readings) < self.number_of_points_for_slope:
            self.logger.info("Too many NaN values encountered")
            self.current_reading = None
            return
        # check if data is too noisy to detect slope and abort if so
        with warnings.catch_warnings():
```

```
        warnings.filterwarnings(
            action="ignore",
            category=ConstantInputWarning,
        )
        res = spearmanr(range(len(readings)), readings).statistic
        # res is nan if all datapoints are the same
    if not np.isnan(res) and abs(res) < 0.5:
        self.logger.info("Data is too noisy to detect slope, aborting...")
        self.exit_flag = True
        return
    # if the above exceptions don't apply, calculate slope
    self.current_reading = abs(
        linregress(
            range(self.number_of_points_for_slope),
            np.array(no_nan_readings[-self.number_of_points_for_slope :])
            / max(no_nan_readings),
        ).slope
    )
```

2.2.2 Calculation of the Jaccard similarity index

Since the similarity index can be reasonably calculated on any kind of two spectra with the same horizontal axis, a helper function was added to the analysis section of the XDL software stack. First, the horizontal axes of the two spectra are aligned to account for digitization error and then a point-by-point comparison is performed to find out the minimum and maximum data point at each horizontal index of the spectrum. These are needed to construct the intersection and union areas later. There is a built-in safety mechanism that equalises the two data points if their difference is smaller than the provided experimental error. This is to account for the mathematically bad conditioning of the minimum(/maximum) function as described in detail in the main manuscript. The algorithm uses a trapezoid integration function imported from the scipy package to calculate the union and intersection areas respectively.

```
def jaccard_two_spectra(
    spectrum_1: AbstractSpectrum,
    spectrum_2: AbstractSpectrum,
    x_precision: int,
    y_error: float,
) -> float:
    """The Jaccard similarity index is a useful metric for comparing two spectra
    when an in-depth analysis of individual peaks is not feasible. The index is
    calculated as the ratio of the intersection area to the union area of the two
    spectra. To obtain these areas, the minimum (/maximum) intensity value of the two
    spectra at each common x-axis point is used to create the intersection (/union)
    spectrum via integration over the common x-axis. To avoid bad conditioning, the
    union and intersection spectra are equalised if the difference in the intensity
    values is smaller than the experimental error y_error.
    """
    common_x_axis = np.round(spectrum_1.x, decimals=x_precision)

    # get y-values of second spectrum at common x-axis
    indices = np.digitize(common_x_axis, spectrum_2.x, right=True) - 1
    indices = np.clip(indices, 0, len(spectrum_2.y) - 1)
    spectrum_2_y = spectrum_2.y.real[indices]
    spectrum_1_y = spectrum_1.y.real
```

```
# create union and intersection of the spectra
union, intersection = [], []
for y1, y2 in zip(spectrum_1_y, spectrum_2_y):
    # equalise union and intersection for small differences to avoid bad conditioning
    if abs(y1 - y2) < y_error:
        union.append(y2)
        intersection.append(y2)
    else:
        union.append(max(y1, y2))
        intersection.append(min(y1, y2))

# calculate Jaccard similarity index
intersection_area = trapezoid(intersection, common_x_axis)
union_area = trapezoid(union, common_x_axis)

return intersection_area / union_area
```

3 Experimental procedures

If not stated otherwise, the following procedure was applied for monitoring experiments:

- The flow cell is primed three times with solvent before acquiring the spectrum.
- The condition of the NMR spectrometer was checked at the beginning of each experiment by acquiring a standard proton spectrum on the solvent(s) that are used on the reaction that is to be monitored.
- Reagents, and solvents are added to the substrate to initiate the reaction.
- Inside the monitoring loop the spectrometer is first shimmed on the strongest signal of the non-deuterated solvent, and then 10 solvent suppression experiments are executed.

The plots of the similarity index and the related metrics can be reproduced from the raw NMR dataset that is shared on the external file sharing system Zenodo. The experiment codes for assigning the datasets are provided in the figure captions.

3.1 Acyclic Terpene precursor under acidic conditions

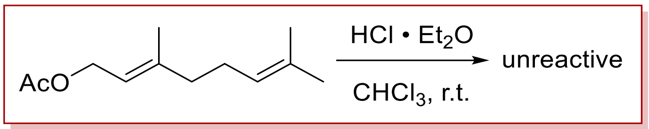

Geranyl acetate (1001.48 mg, 1093.32 µL, 5 mmol, 1 equiv.) was dissolved in 50 mL $CHCl_3$. Then, HCl in diethyl ether (5 mL, 1 mol/L, 5 mmol, 1 equiv.) was added to the mixture. Monitoring was conducted for a total amount of 1 d 4 h 13 min 19 s. No significant reaction was observed during that time. This can be confirmed by the Spearman correlation being close to zero, which indicates that the data has no clear trend in any direction.

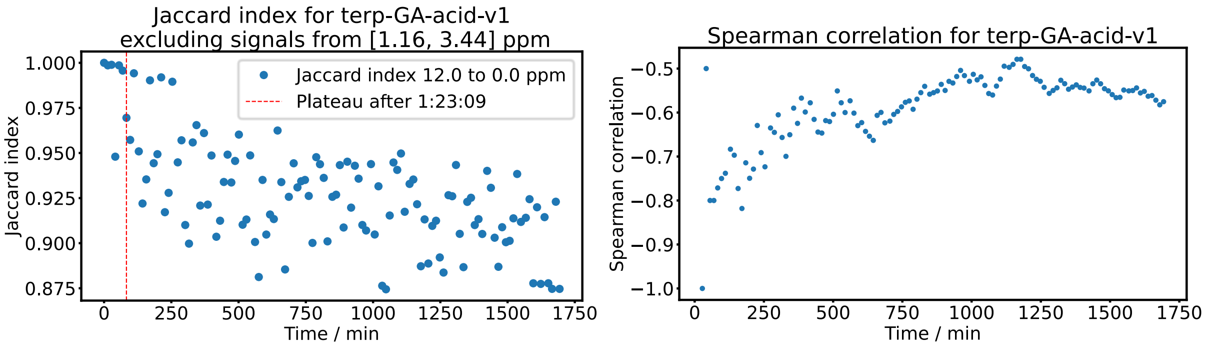

Figure Ex1: terp-GA-acid-v1. Similarity data from NMR monitoring

3.2 Formose reaction

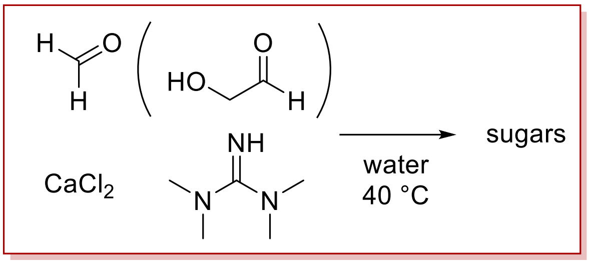

Aqueous stock solutions of all reagents were prepared in advance: formaldehyde (1 mol/L), glycolaldehyde dimer (0.2 mol/L), tetramethyl guanidine (1 mol/L), $CaCl_2$ (0.02 mol/L). 5 mL of each stock solution were added to the reactor flask, and the hotplate was set to 40 °C to commence the reaction. Two experiments were set up: one batch without the glycolaldehyde dimer that acts as an auto catalyst where 5 mL of water were added instead and one batch with the catalyst. Monitoring of the first batch (without glycolaldehyde dimer) was conducted for a total time of 15 h 44 min 56 s without significant reaction progress.

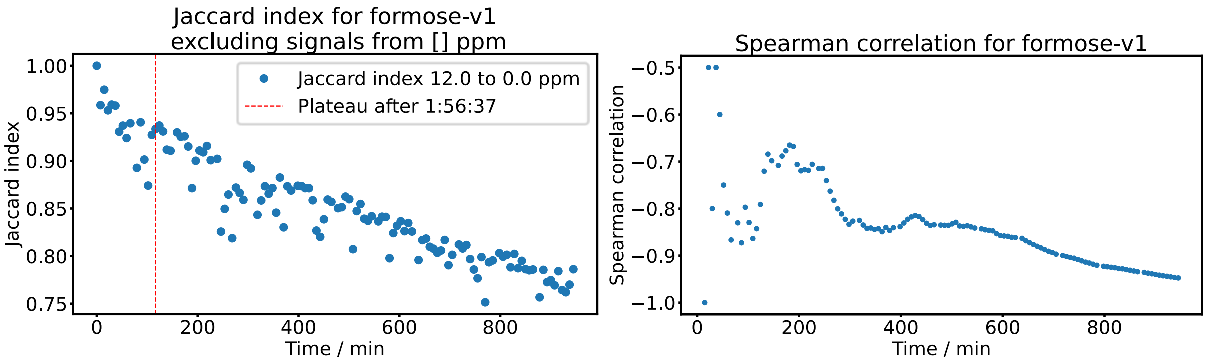

Figure Ex2.1: formose-v1. Similarity data from NMR monitoring

Monitoring of the second batch (with glycolaldehyde dimer) was conducted for a total time of 23 h 25 min 09 s.

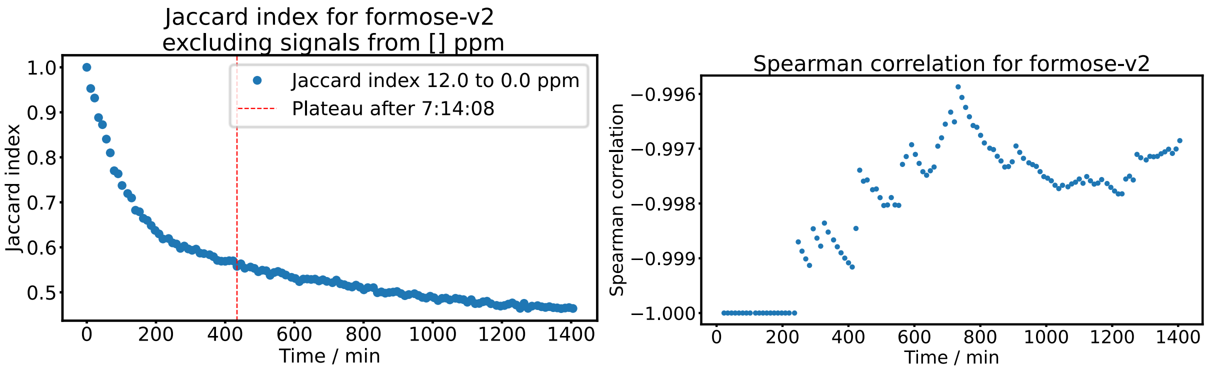

Figure Ex2.2: formose-v2. Similarity data from NMR monitoring

3.3 Maillard reaction

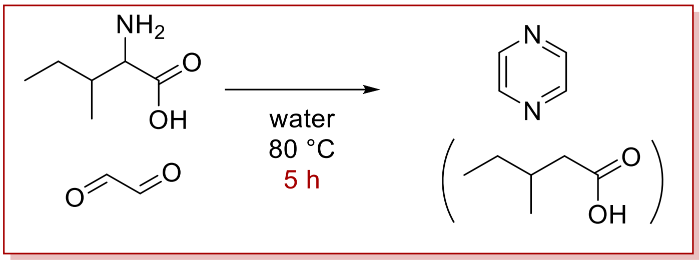

Isoleucine (393.51 mg, 3 mmol, 1 equiv.), glyoxal (435.30 mg, 344 µL, 3 mmol, 1 equiv.), and NaOH (119.99 mg, 3 mmol, 1 equiv.) were added to the reactor flask and dissolved in 10 mL of water. The hotplate was set to 80 °C to commence the reaction. Monitoring was conducted for a total amount of 17 h 14 min 51 s. The reaction was found to have reached a stable state after 4 h 53 min.

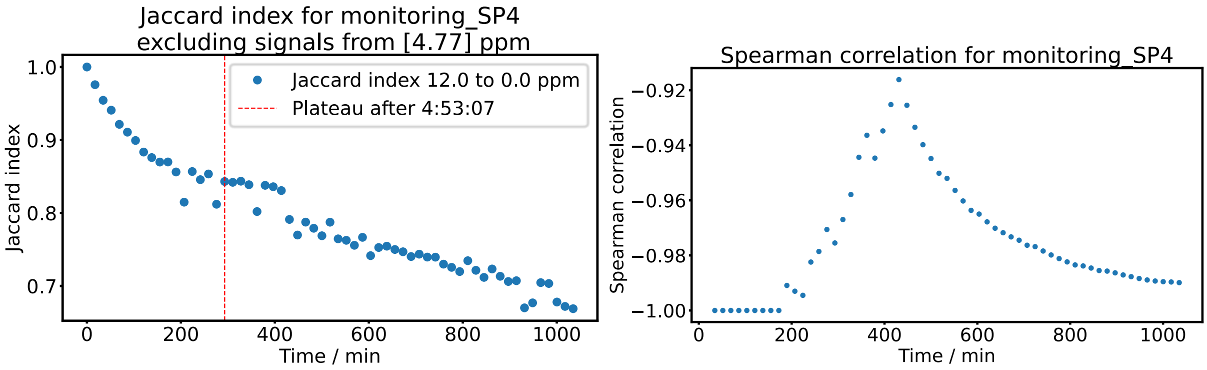

Figure Ex3: monitoring_SP4. Similarity data from NMR monitoring

3.4 Imine formation

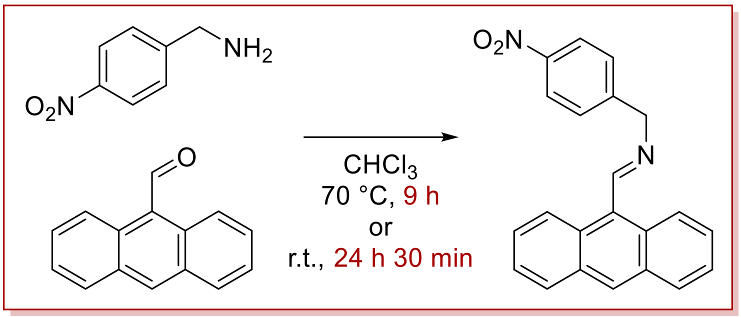

4-Nitrobenzylamine hydrochloride (601.95 mg, 3 mmol, 1 equiv.) and 9-Anthracenecarboxaldehyde (637.86 mg, 3 mmol, 1 equiv.) were dissolved in 20 mL of $CHCl_3$. Two batches were prepared: the first one was monitored at room temperature for a total time of 1 day 9 h 50 min 32 s. The reaction was found to have reached a stable plateau after 24 h 28 min.

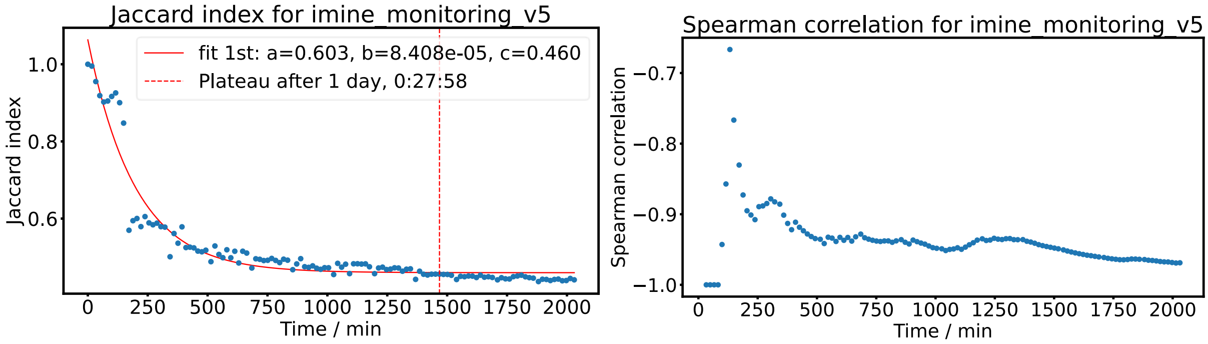

Figure Ex4.1: imine_monitoring_v5. Similarity data from NMR monitoring

The second one was monitored at 70 °C for a total time of 1 day 2 min 32 s. The reaction was found to have reached a stable state after 9 h.

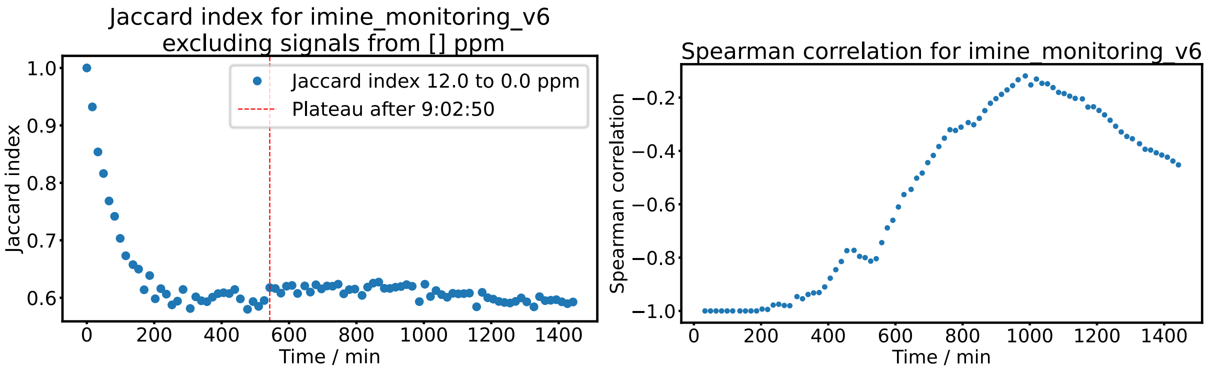

Figure Ex4.2: imine_monitoring_v6. Similarity data from NMR monitoring

3.5 Photochemical debromination

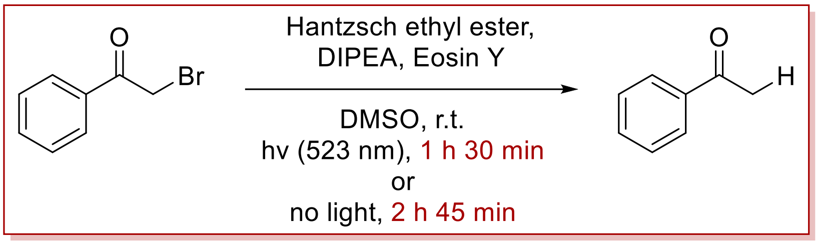

2-bromo-1-phenylethan-1-one (199.05 mg, 1 mmol, 1 equiv.), diethyl 2,6-dimethyl-1,4-dihydropyridine-3,5-dicarboxylate (278.62 mg, 1.1 mmol, 1.1 equiv.), Eosin Y (16.20 mg, 0.1 mmol, 0.2 equiv.), degassed dimethyl sulfoxide (16 mL) and N,N-diisopropyl ethylamine (258.49 mg, 348 µL, 2 mmol, 2 equiv.) were added to a 20 mL vial. Two batches were prepared: the first one was irradiated at 523 nm with a photoreactor and monitored for a total amount of 5 h 12 min 40 s. The reaction was found to have reached a stable state after 1 h 30 min (not indicated in the plot because the platform terminated the monitoring and initiated the workup sequence right after detecting the plateau).

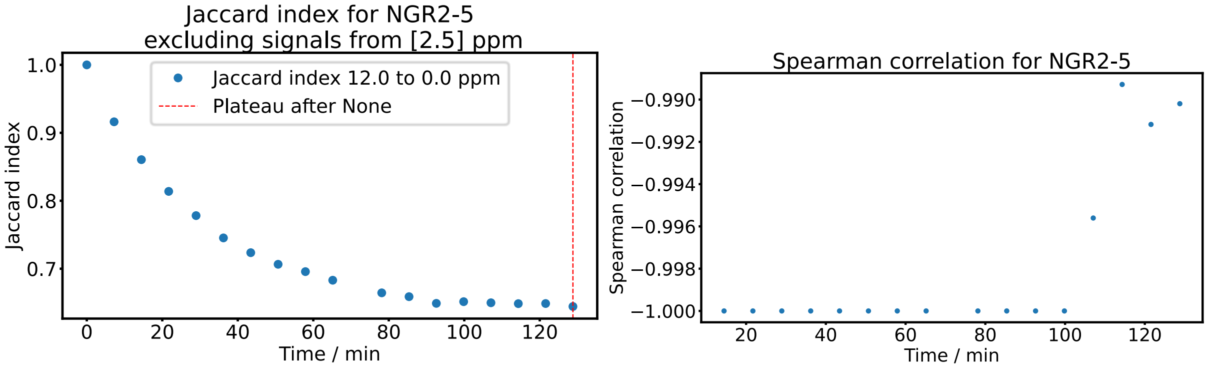

Figure Ex5.1: NGR2-5. Similarity data from NMR monitoring

The second batch was not irradiated with a photoreactor but was still exposed to ambient light.

The reaction was monitored for 22 h 16s and reached a stable state after 2 h 45 min.

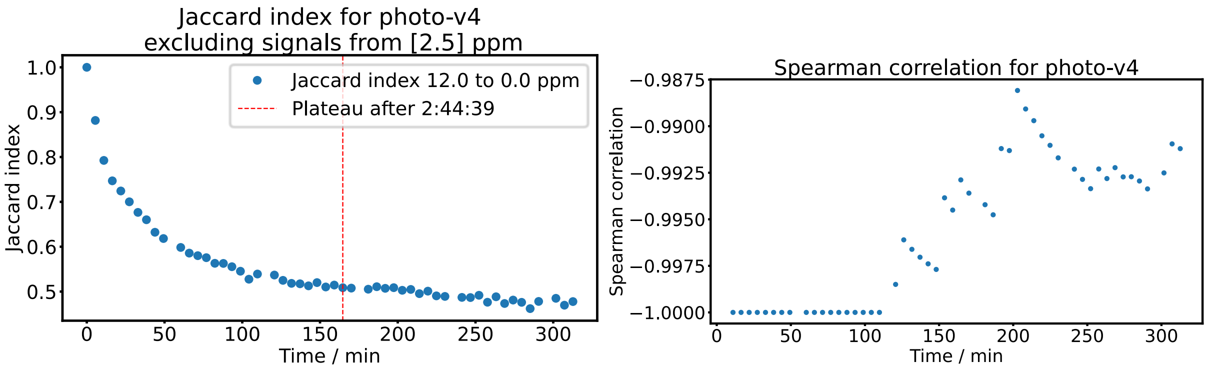

Figure Ex5.2: photo-v4. Similarity data from NMR monitoring

3.6 Formation of Lophine

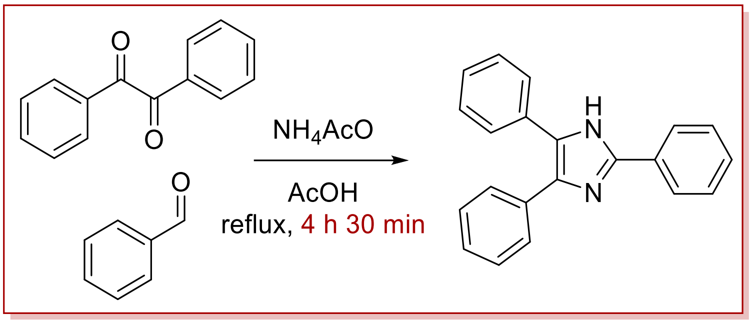

Benzil (318.53 mg, 1.5 mmol, 1 equiv.), benzaldehyde (525.29 mg, 502 µL, 4.95 mmol, 3.3 equiv.), and ammonium acetate (1769.69 mg, 22.50 mmol, 15 equiv.) were dissolved in glacial acetic acid (50 mL) and the hotplate was set to 140 °C to achieve reflux. The reaction was monitored for 18 h 46 min 42 s and reached a stable state after 4 h 35 min.

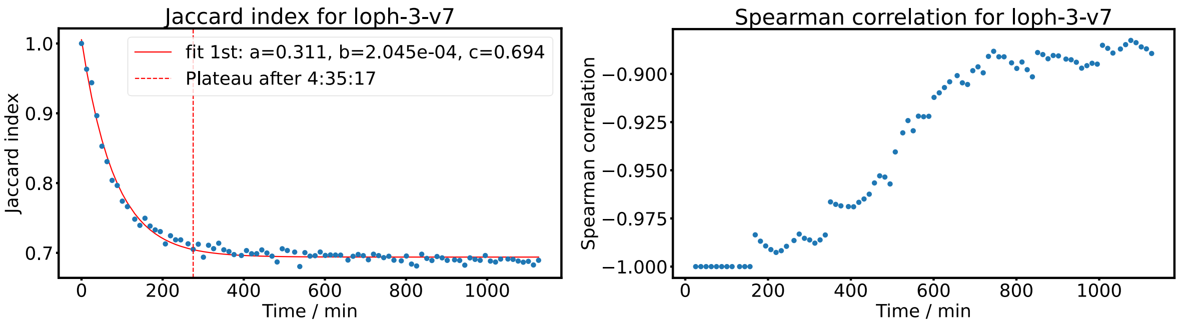

Figure Ex6: loph-3-v7. Similarity data from NMR monitoring

3.7 Buchwald-Hartwig amination

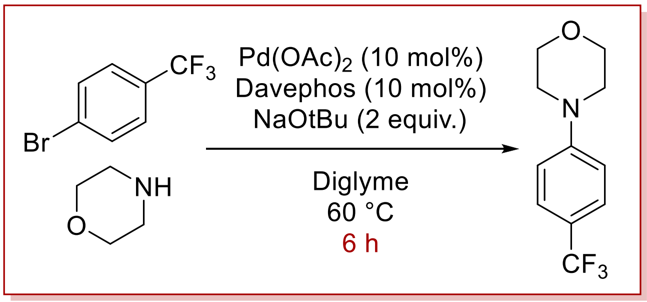

An oven-dried 3-neck RBF (100 mL) was charged with a sodium tert-butoxide (776.61 mg, 8 mmol, 2 equiv.), DavePhos (162.29 mg, 0.4 mmol, 0.1 equiv.), $Pd(OAc)_2$ (89.804 mg, 0.4 mmol, 0.1 equiv.), 4-bromobenzotrifluoride [by syringe] (0.57 mL, 4 mmol, 1.0 equiv.). The reactor was evacuated for 1 min and refilled with argon for three times. Then, diglyme (20 mL) and morpholine (0.52 mL, 6 mmol, 1.5 equiv.) were added. The flask was purged with Argon for 5 minutes; the hotplate was set to 60 °C and the reaction was monitored for 23 h 17 min 40 s. In each monitoring cycle, a ^{1}H (with solvent suppression) and a ^{19}F spectrum were acquired and the two sets of spectra were analysed separately. Both the ^{1}H and the ^{19}F spectra indicated that the reaction reached a stable state after around 6 h 15 min.

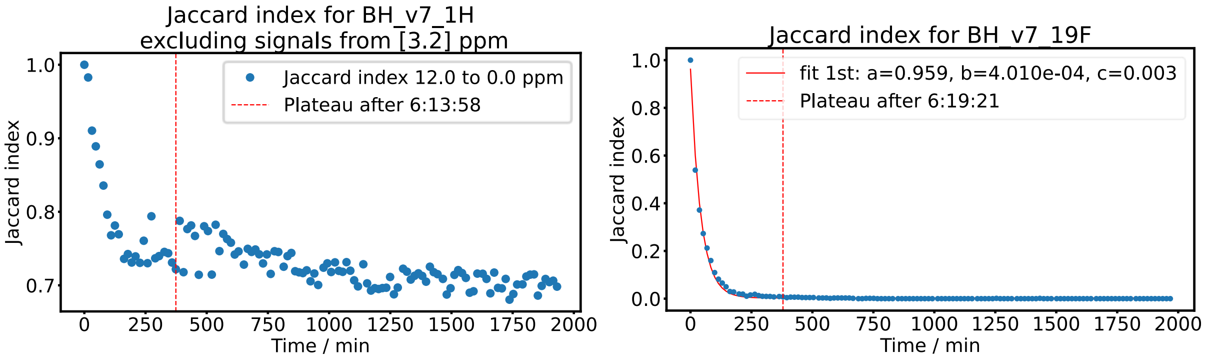

Figure Ex7: BH_v7_1H & BH_v7_19F. Similarity data from NMR monitoring

After cooling the reaction was stopped by quenching with an aqueous saturated NH_4Cl solution (30 mL) yielding in a suspension. The organic layer was extracted with CH_2Cl_2 (3x15 mL), and the combined organic layers were washed with saturated NH_4Cl solution (20 mL). The separation was very slow, and it took around 5 min to separate the layers. The combined organic layers were dried over Na_2SO_4 and CH_2Cl_2 was evaporated at 600 mbar and 40 °C. The residual 20 mL of diglyme were removed by vacuum distillation at 5 mbar and 70 °C hotplate temperature (40 °C head temperature). The crude product (brown liquid) was injected onto a flash chromatography silica column (25 g) and eluted with hexanes/ethyl acetate (90/10) to give a pure product as a beige crystalline solid (232.2 mg, 9.9 %).

3.8 Photochemical oxidation of 9,10-Diphenylanthracene

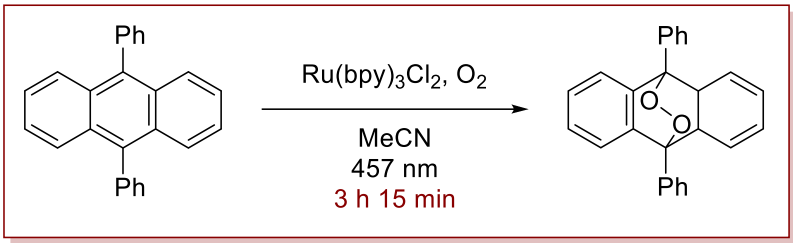

Reaction conditions were adapted from Pitre *et al.* [6] and modified to accommodate the constraints of the Chemputer platform. 9,10-diphenylanthracene (6.7 mg, 20.3 µmol, 1.00 equiv.) and tris(2,2'-bipyridyl)dichlororuthenium(II) hexahydrate (30.2 mg, 40.3 µmol, 1.99 equiv.) were added to an oven-dried Duran flask. The flask was placed under vacuum and backfilled with nitrogen three times. The reagent and the catalyst were dissolved in acetonitrile (400 mL) in the dark. The UV/Vis spectra of this stock solution and of pure acetonitrile were measured. Aliquots of the stock solution (15 mL) were transferred to 20 mL vials in the photoreactor to be irradiated under stirring at full irradiation intensity and maximum cooling power with 457 nm light in 1 s intervals for a total of 25 s. In between each irradiation interval, aliquots of the reaction mixtures (2.5 mL) were analysed using UV/Vis spectroscopy. After each measurement, the aliquot was transferred back to its vial.

Application of the Jaccard similarity index calculation yielded a smooth data series, which reached a stable plateau after around 3 h 15 min as seen in Figure Ex8.

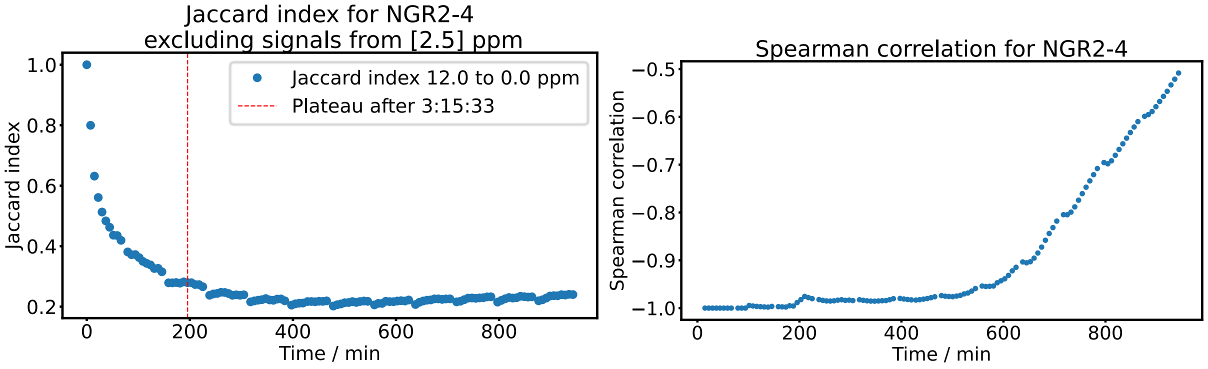

Figure Ex8: NGR2-4. Jaccard similarity index for the UV/Vis spectra of the photochemical oxidation of 9,10-Diphenylanthracene. Analogously to the previously presented NMR data, the similarity of the UV/Vis spectra reaches a stable plateau when the reaction is finished, and the spectrum does not change any more.

3.9 The reaction between potassium permanganate and oxalic acid

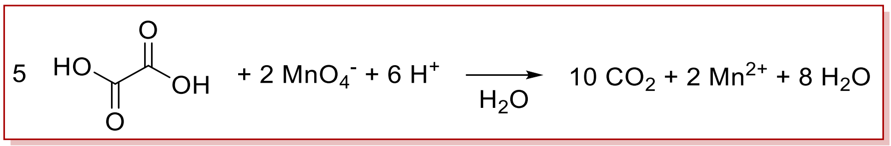

Potassium permanganate (3.23 mg, 0.02 mmol, 2 equiv.) oxalic acid (6.43 mg, 0.05 mmol, 5 equiv.) and sulfuric acid (3.0 mg, 2 μL, 0.03 mmol, 3 equiv.) were dissolved in water (20 mL) and the reaction mixture was stirred while an assembly of syringe pumps continuously moved liquid from the mixture through the flow cell of a UV/Vis spectrometer for rapid sampling in intervals of 200 milliseconds. The resulting spectral data was analysed with *via* the Jaccard similarity index to give a sigmoidal shaped series that was compared against the result from kinetic simulations based on a model from Adler and Noyes.

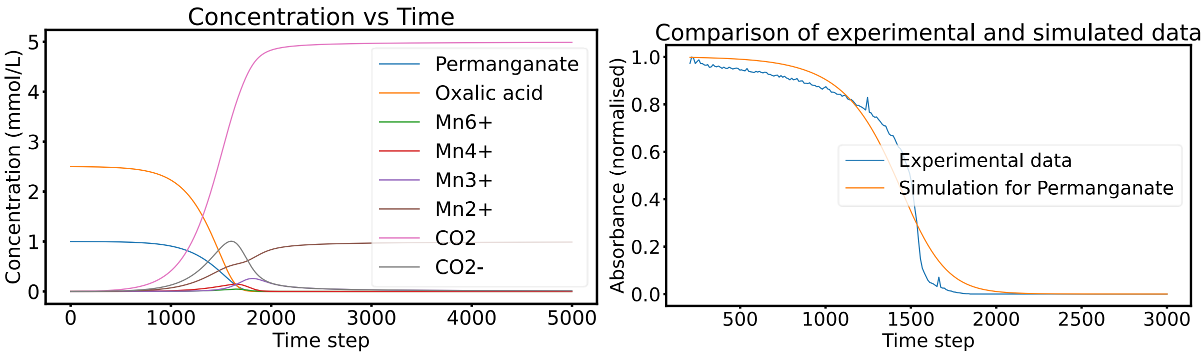

Figure Ex9: perm_v2. Comparing a numerical simulation of the permanganate-oxalate-reaction with experimental UV/Vis data. Based on a mechanistic model from Adler and Noyes [7] a system of elementary reactions was derived and first order kinetics were assumed for all reagents for reasons of simplicity. The resulting rate equations were then used to run a numerical simulation for the concentration profiles of all reagents (left-hand side). The concentration profile of permanganate was then compared against experimental data because permanganate is the species with the most prominent signal in the UV/Vis range (right-hand side).

3.10 Belousov-Zhabotinsky reaction

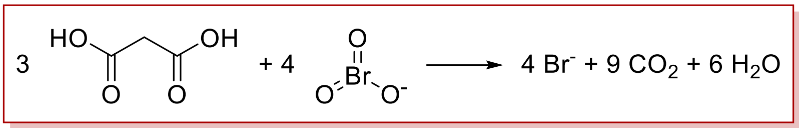

Aqueous stock solutions of all reagents were prepared as follows:

- Malonic acid (10.406 g, 100 mmol) in water (100 mL) to give 1.0 mol/L
- Potassium bromate (7.174 g, 25 mmol) in 1 M sulfuric acid (50 mL) to give 0.5 mol/L
- Sulfuric acid (9.807 g, 100 mmol) in water (100 mL to give 1.0 mol/L
- Iron (II) sulphate heptahydrate (27.8 mg, 0.1 mmol, 1 equiv.) and 1,10-phenanthroline (54.06 mg, 0.3 mmol, 3 equiv.) in water (10 mL) to give 0.01 mol/L of ferroin indicator

Subsequently, malonic acid (9 mL, 1.0 M, 9 mmol, 1 equiv.), sulfuric acid (6.25 mL, 1.0 M, 6.25 mmol), potassium bromate (9.5 mL, 0.5 M, 4.75 mmol, 0.53 equiv.), ferroin indicator (0.625 mL, 0.01 M, 6.25 µmol, 7e-4 equiv.), and water (9.625 mL) were mixed in a 50 mL round bottom flask. The reaction mixture was stirred while an assembly of syringe pumps continuously moved liquid from the mixture through the flow cell of a UV/Vis spectrometer for rapid sampling in intervals of 200 milliseconds. The resulting spectral data was analysed with *via* the Jaccard similarity index to give an oscillating pattern that was subjected to Fourier transformation. The dominating frequency was found to be 0.138 Hz corresponding to an oscillation interval of 7.25 seconds.

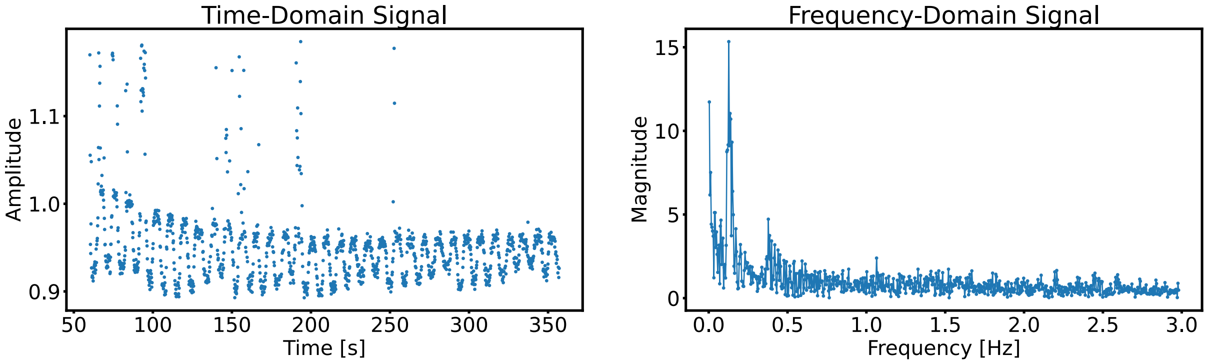

Figure Ex10: BZ_v3. Jaccard similarity index series and Fourier transformation of the same for a Belousov-Zhabotinsky reaction. Following the reaction with UV/Vis spectroscopy in very short intervals of 200 milliseconds allows to extract the chemical oscillation from the spectroscopic data by the means of similarity analysis.

3.11 Photochemical excitation of a naphthalene diamide (NDI) gel

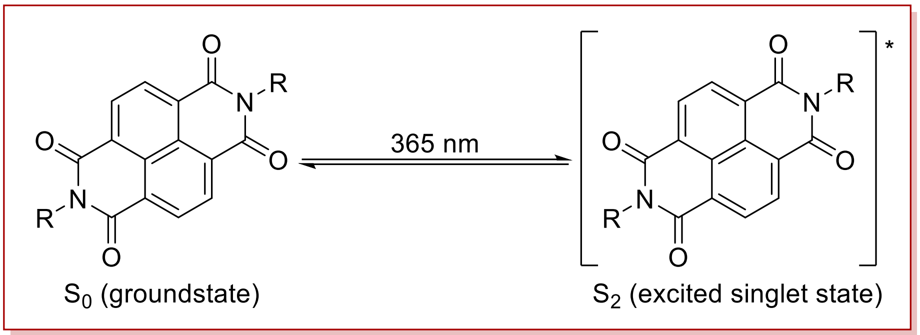

NDI was obtained from collaborators according to synthetic procedures described by Randle *et al.* [8] and a solution of 10 mg/mL was prepared by dissolving NDI in 2 molar equivalents of aqueous NaOH (0.1 M) and 400 µL/mL of 0.1 M NaCl (as background electrolyte). The mixture was diluted with deionised water to reach the desired concentration. The solution was stirred overnight until all solids had dissolved.

The solution was then monitored *via* EPR spectroscopy while being irradiated at 365 nm. The spectra were integrated to obtain absorption-mode signals before applying the similarity algorithm to follow the process.

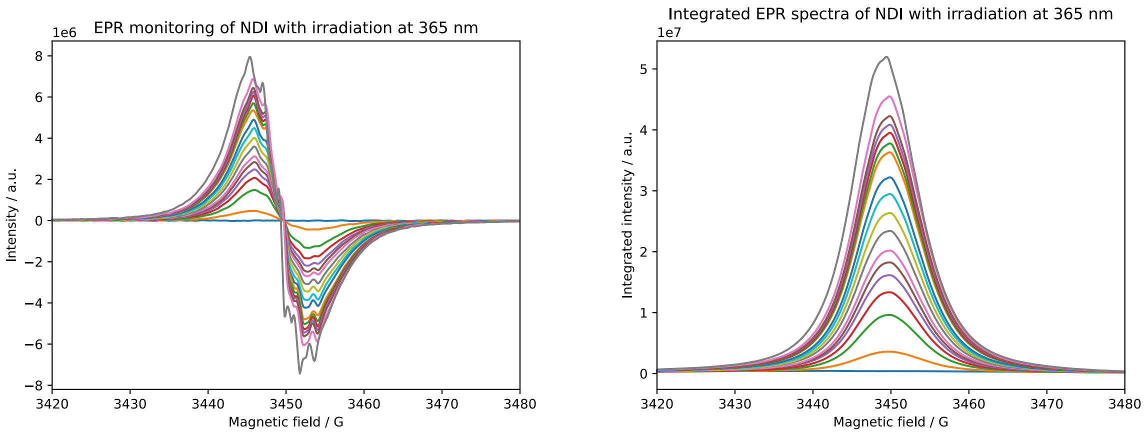

Figure Ex11.1: NDI_monitoring. EPR monitoring of the photochemical excitation of an NDI gel. The original data (left) was integrated to obtain absorption-mode signals.

It was found that comparing against the final spectrum in the series is most insightful in this case because there is no signal at the beginning of the irradiation due to the absence of an excited paramagnetic state. Comparing against a flat baseline will always return a substantial dissimilarity and would render the comparison trivial.

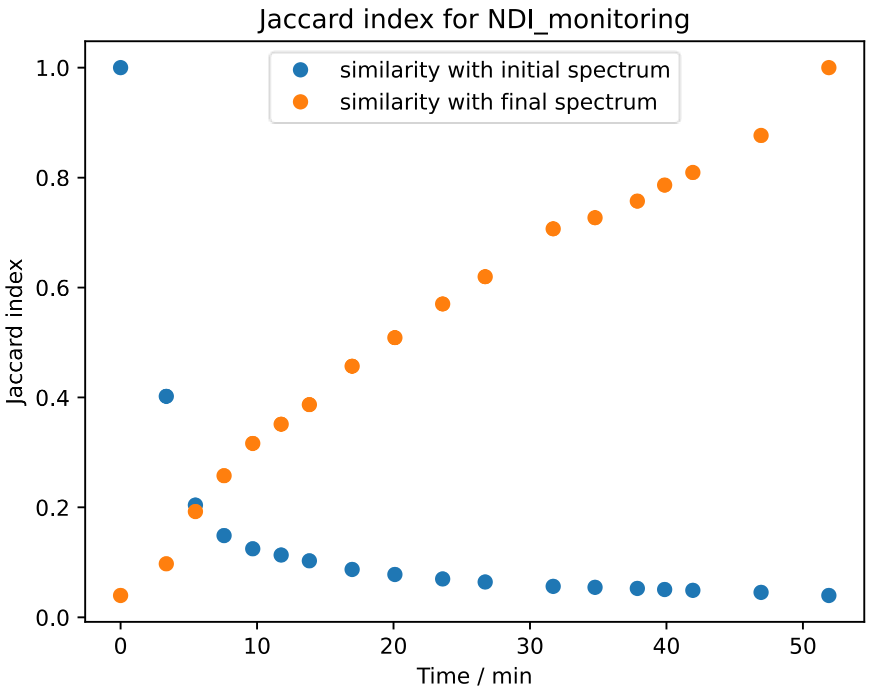

Figure Ex11.2: NDI_monitoring. Jaccard similarity index between EPR spectra over time. For blue line all spectra were compared to the first spectrum in the dataset, for the orange line last spectrum was taken as a reference.

3.12 Azide formation

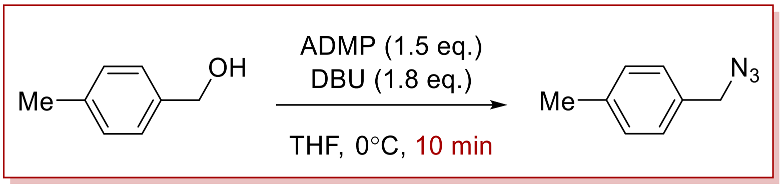

To a solution of 4-methylbenzyl alcohol (190 mg, 1.56 mmol, 1.0 equiv.) in tetrahydrofuran (30 mL) was added 2-azido-1,3-dimethylimidazolinium hexafluorophosphate (ADMP) (710 mg, 2.49 mmol, 1.6 equiv.) and the mixture was cooled to 0 °C. 1,8-Diazabicyclo(5.4.0)undec-7-ene (DBU) (426 mg, 0.42 mL, 2.80 mmol, 1.8 equiv.) was added dropwise to initiate the reaction.

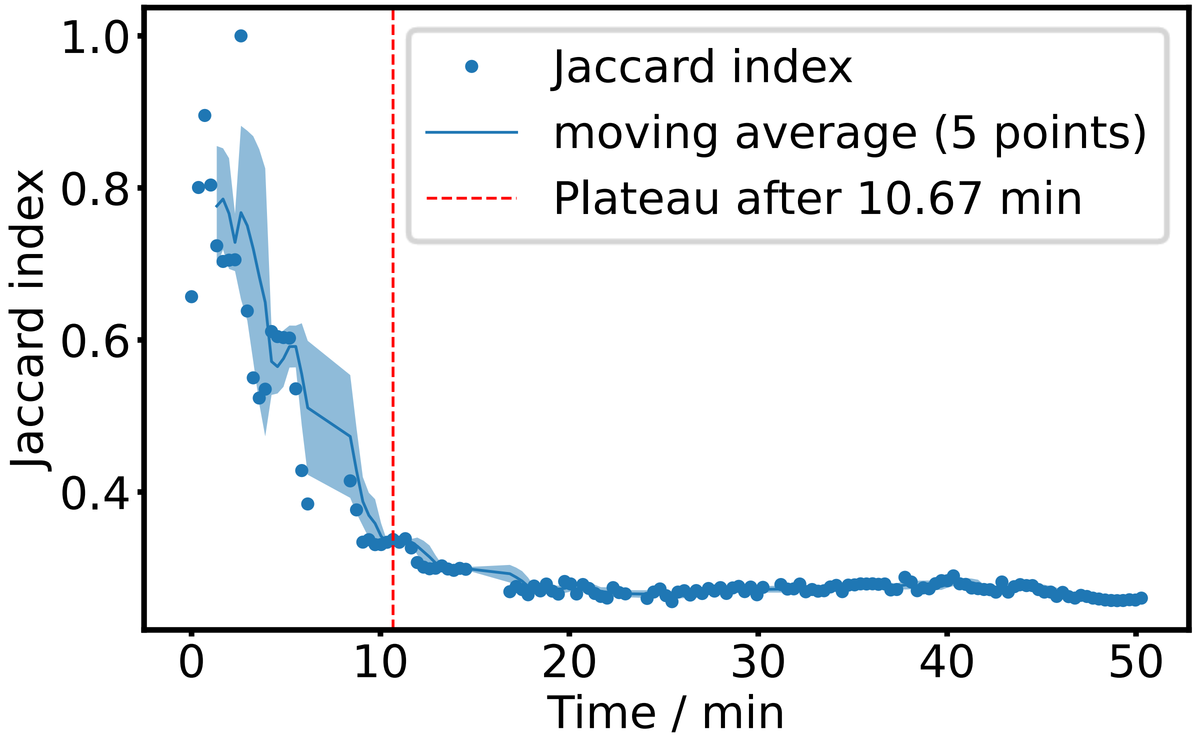

Figure Ex12: azide_v2. Similarity data from IR monitoring

3.13 Molecular switching of a rotaxane

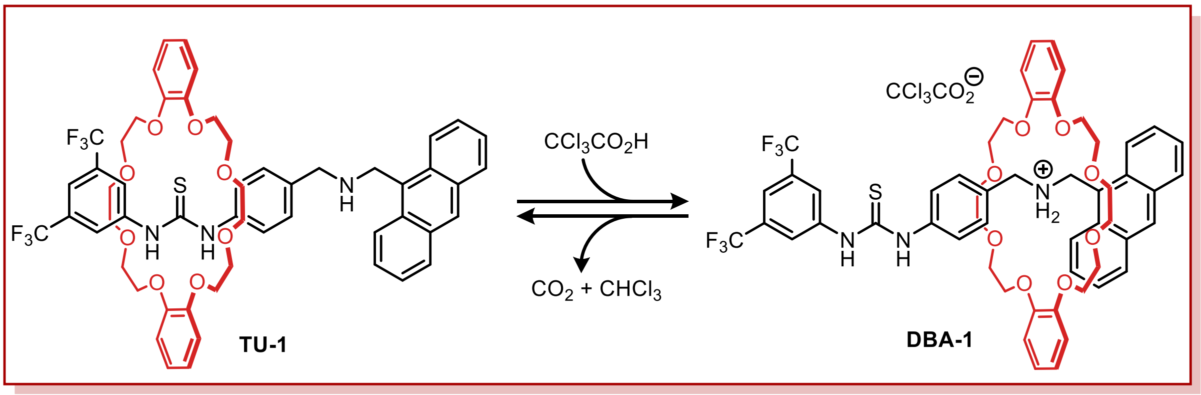

DBA-1 was synthesized in its protonated form with trifluoroacetate as counterion according to a previously published protocol [9] and an aliquot (62 mg, 0.06 mmol, 1 equiv.) was dissolved with deuterated chloroform (0.6 mL) in an NMR tube. DBU (19 mg, 18 µL, 0.12 mmol, 2 equiv.) was added to the NMR tube to ensure that the rotaxane is fully deprotonated, which was confirmed through the disappearance of the benzylic proton signals from the NMR spectrum. Subsequently, trichloroacetic acid (TCA) (20 mg, 0.12 mmol, 2 equiv.) was added to the NMR tube, the tube was vortexed to ensure mixing and placed in the NMR spectrometer for monitoring. Spectra were acquired in intervals of 2 minutes.

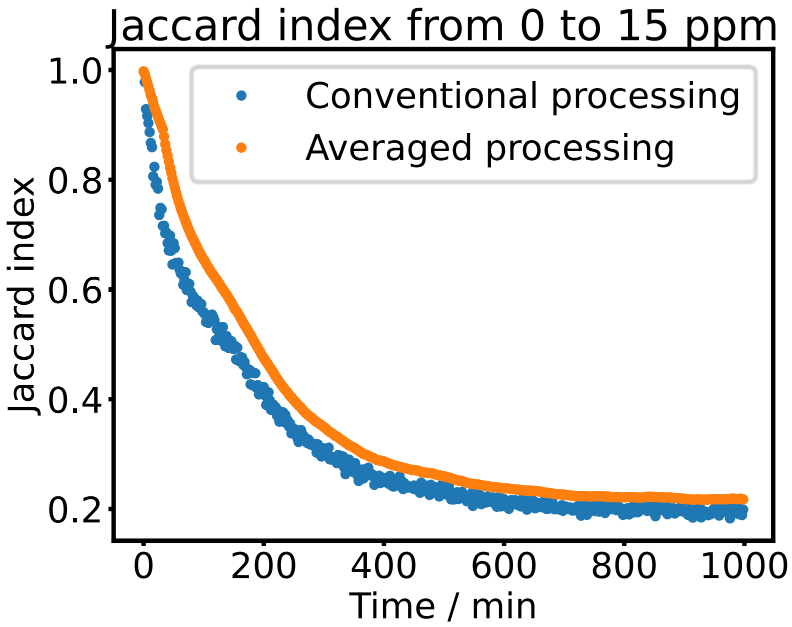

Figure Ex13: RR-M3-5bb-moni-v3. Similarity data from NMR monitoring

4 Reaction monitoring *via* conversion tracking

To validate the results from the monitoring with the Jaccard similarity index, the formation of Lophine was monitored in a traditional way by picking diagnostic signals in the assigned NMR spectrum and calculating the conversion from them. The time after which the conversion reached a plateau was later compared to the time after which the Jaccard index reached a stable plateau, and the time points were found to be in good agreement.

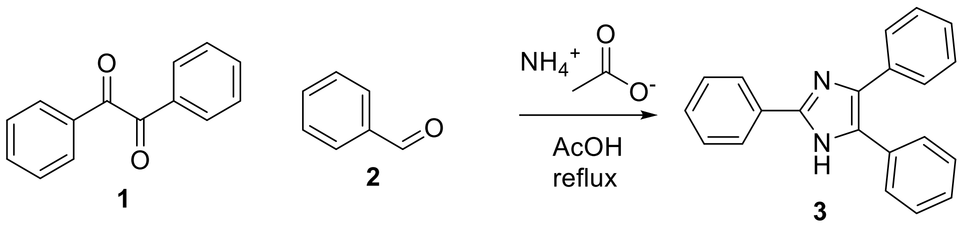

The raw spectra were processed as follows:

- Apodization (function=”gm”, g1=1.2, g2=4.5)
- Zero filling (doubling the length of the FID)
- Fourier transformation
- Referencing to 1.8 ppm (the acetic acid signal, which is the strongest signal in the spectrum)
- Integration of the two characteristic peaks:
    - From 7.7 to 7.5 ppm
    - From 7.5 to 7.1 ppm

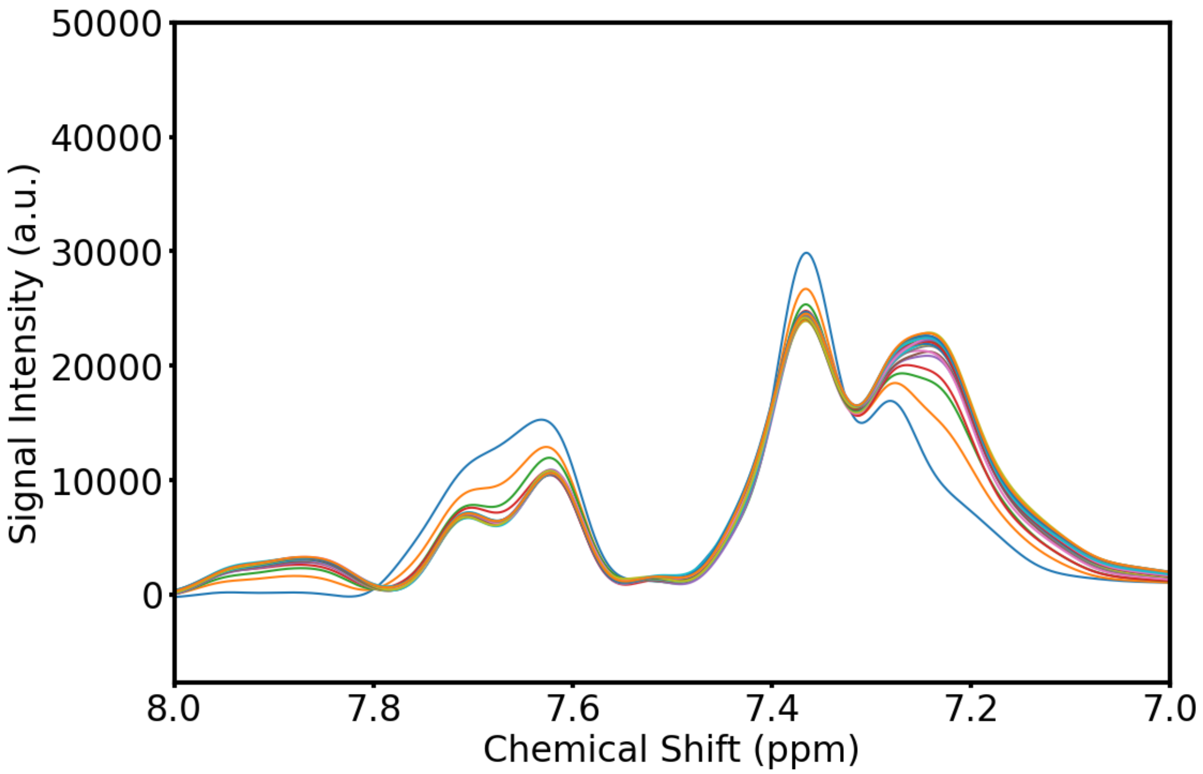

Figure S2: Diagnostic window in the NMR spectrum during the formation of lophine.

The signals in the aromatic region were used to calculate the conversion X according to Equation S1 as the ratio of the product integral $I_{product}$ divided by the sum of the integrals of product and starting material $I_{substrate}$. In this case, the signals used correspond to the protons in meta position on the phenyl groups of benzil (compound **1**) and the corresponding positions in Lophine (compound **3**). The obvious use of the aldehyde signal as a diagnostic measure was not possible in this case because it is overlaid by the broad signal of the acidic proton of the acetic acid that is used as a solvent.

$$X = \frac{I_{\text{product}}}{I_{\text{substrate}} + I_{\text{product}}} \tag{S1}$$

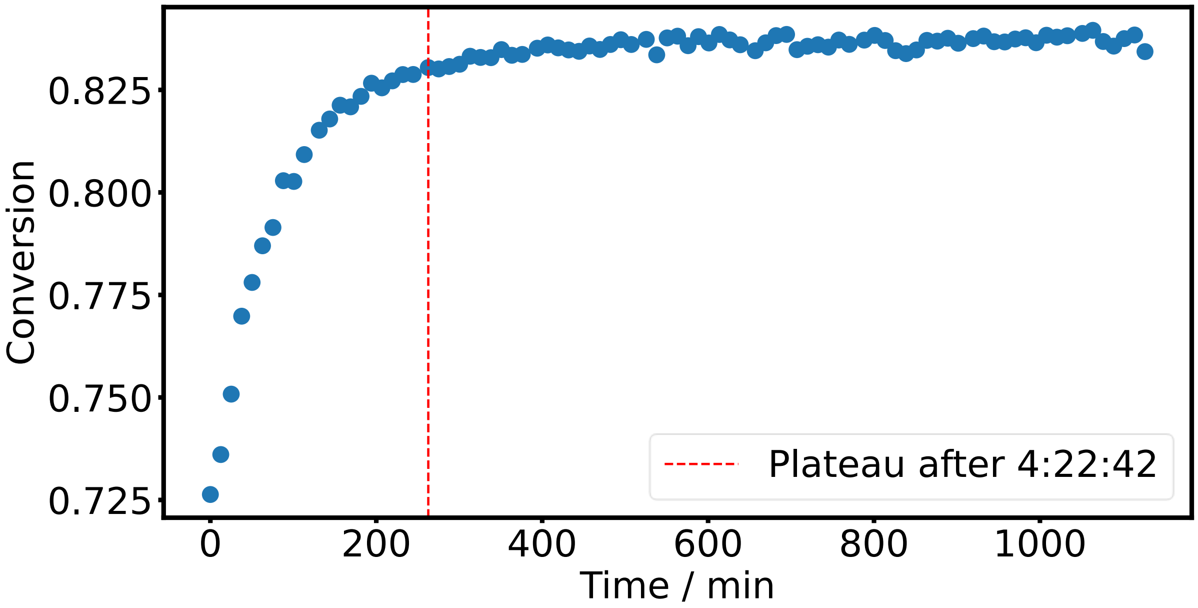

Figure S3: **Conversion time series for the formation of lophine.** The conversion X was calculated by comparing the characteristic signals in the aromatic region.

5 Spearman correlation coefficient to detect noisy data

When monitoring a blank solvent sample without any reactive chemicals, one would intuitively expect the Jaccard similarity index to adopt a stable plateau around 1.0 immediately after starting the experiment. Contrary to the expectations, the Jaccard data for an unreactive mixture (Figure S4) was not found to settle in an early plateau; instead, there were strong fluctuations in the calculated Jaccard index leading to an erratic behaviour of the plateau-detection algorithm. As a first remedy to this behaviour, the course of the Jaccard data was analysed using Spearman's rank correlation coefficient [10] as defined in Equation S2.

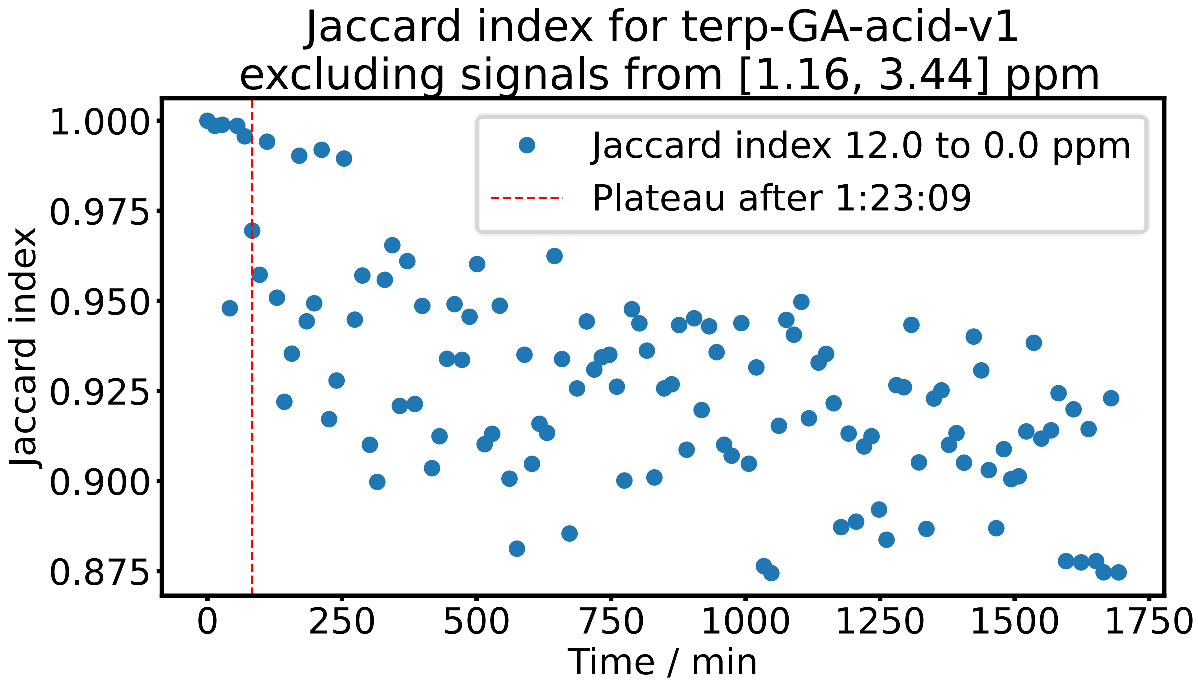

Figure S4: **Evolution of the Jaccard similarity index for an unreactive mixture.** Intuitively, the similarity index would be expected to adopt a stable plateau immediately because there is no chemical reaction that would change the composition of the spectrum. Instead, the data is unusually noisy.

$$r_s = \frac{\mathrm{cov}\big(R(X), R(Y)\big)}{\sigma_{R(X)} \cdot \sigma_{R(Y)}} \qquad (S2)$$

cov ≔ covariance

$R(X) \coloneqq$ rank variables for dataset X

$\sigma_{R(X)} \coloneqq$ standard deviation of the rank variables for dataset X

Spearman's rank correlation coefficient determines if the data is broadly following a monotonic trend (which would be indicated by an absolute rank correlation close to one) or rather if it is undergoing random fluctuations (indicated by a rank correlation coefficient around zero). When detecting a very low rank correlation, the experiment can be automatically aborted by the control software, assuming that the data is either corrupted or there is no reaction happening. Figure S5 shows how the rank correlation coefficient remains close to −1.0 for a monotonically decaying Jaccard index.

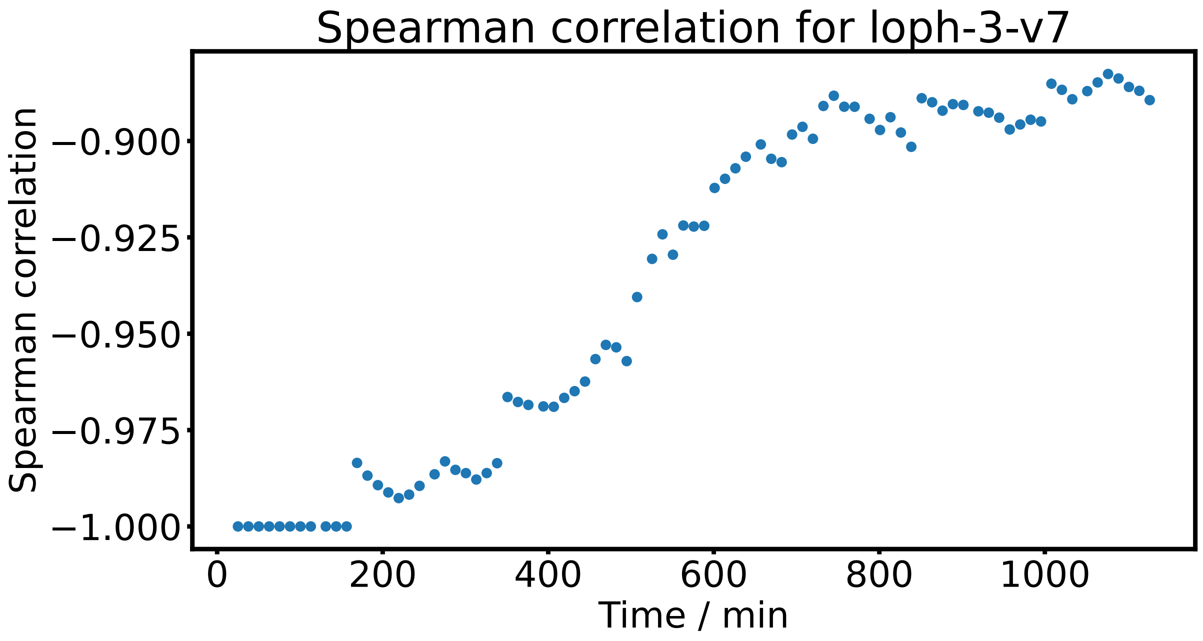

Figure S5: **Monitoring results for the formation of Lophine obtained from an 80 MHz bench-top NMR spectrometer.** The Spearman correlation coefficient of the Jaccard similarity values to attest smooth reaction progress.

6 Numerical analysis of the Jaccard algorithm

Ultimately, the fluctuation of the Jaccard index must be related to the experimental error of the NMR measurement, which is the main source of noise in the whole process. In numerical mathematics there are different characteristic values that can be considered when analysing the influence of a disturbed input ($\tilde{x}$) on a mathematical problem ($f(\boldsymbol{x})$) that depends on the true input ($\boldsymbol{x}$) and is approached with a numerical algorithm ($\tilde{f}$). These characteristic properties include:

- $\|f(\tilde{x}) - f(x)\|$ condition: How much does the problem vary when disturbed?
- $\|\tilde{f}(\tilde{x}) - \tilde{f}(x)\|$ stability: How much does the numerical algorithm vary with disturbance?
- $\|\tilde{f}(x) - f(x)\|$ consistency: How well does the algorithm solve the actual problem when given the true input?
- $\|\tilde{f}(\tilde{x}) - f(x)\|$ convergence: How well does the disturbed algorithm solve the actual problem?

When thinking about the algorithm for the calculation of the Jaccard index, the critical step is hidden in the comparison of the intensity values because these carry the experimental error from the machine into the spectrum. Consequently, the numerical analysis of the overall process is mostly correlated to the minimum (or maximum) function applied to calculate the intersection (or union) of two spectra. The minimum function is a mathematically exact operation, so the numerical approximation $\tilde{f}$ does not need to be considered; here, only the condition of the problem is relevant.

The absolute condition number κ_{abs} is defined as the multiplication factor between the input error $\|\tilde{\mathbf{x}} - \mathbf{x}\|$ and the upper bound for the output error of the problem.

$\mathbf{x} :=$ true input

$\tilde{\mathbf{x}} :=$ disturbed input

$f(\mathbf{x}) : \mathbb{C}^n \rightarrow \mathbb{C}^m$ a mathematical transformation

$$\|f(\tilde{\mathbf{x}}) - f(\mathbf{x})\| \leq \kappa_{\text{abs}} \cdot \|\tilde{\mathbf{x}} - \mathbf{x}\|$$

(1)

In mathematical terms, this can be equivalently expressed as a limit superior.

$$\kappa_{\text{abs}} = \lim_{\tilde{\mathbf{x}} - \mathbf{x}} \sup \frac{\|f(\tilde{\mathbf{x}}) - f(\mathbf{x})\|}{\|\tilde{\mathbf{x}} - \mathbf{x}\|}$$

(2)

Now, consider the minimum function $f(\mathbf{x}) = \min(x_1, x_2)$, which has been identified to be crucial to the calculation of the Jaccard similarity index, as the mathematical transformation f.

$$\kappa_{\text{abs}} = \lim_{\tilde{\mathbf{x}} - \mathbf{x}} \sup \frac{\|\min(\tilde{x}_1, \tilde{x}_2) - \min(x_1, x_2)\|}{|\tilde{x}_1 - x_1| + |\tilde{x}_2 - x_2|}$$

(3)

The evaluation of the minimum function is case dependant on the comparison of the two input values x_1 and x_2. Four possible cases can be considered.

1. $x_1 > x_2, \tilde{x}_1 > \tilde{x}_2$
2. $x_1 > x_2, \tilde{x}_1 \leq \tilde{x}_2$
3. $x_1 \leq x_2, \tilde{x}_1 > \tilde{x}_2$
4. $x_1 \leq x_2, \tilde{x}_1 \leq \tilde{x}_2$

First, consider case 1, for which the minimum function is evaluated as $\min(x_1, x_2) = x_2$. By using the taxicab norm $\|\tilde{\mathbf{x}} - \mathbf{x}\| = |\tilde{x}_1 - x_1| + |\tilde{x}_2 - x_2|$ the expression for the condition number κ can be simplified leading to an upper bound of one. This is interpreted as no error amplification between the input and the output error, which means there are no issues in this case.

$$\kappa_{\text{abs}} = \limsup_{\tilde{\mathbf{x}}-\mathbf{x}} \frac{|\tilde{x}_2 - x_2|}{|\tilde{x}_1 - x_1| + |\tilde{x}_2 - x_2|}$$

$$= \limsup_{\tilde{\mathbf{x}}-\mathbf{x}} \frac{1}{\frac{|\tilde{x}_1 - x_1|}{|\tilde{x}_2 - x_2|} + 1}$$

$$= 1$$

Next, case 2 is considered, which is more complicated because the disturbance of the input now changes which of the two values is smaller. Using the triangle inequality $x + y \leq |x| + |y|$ allows to simplify the expression but, in the end, the term in the nominator does not vanish. Therefore, evaluating the limit superior for $\tilde{x} \to x$ leads to an unbound expression.

$$\kappa_{\text{abs}} = \limsup_{\tilde{\mathbf{x}}-\mathbf{x}} \frac{|\tilde{x}_1 - x_2|}{|\tilde{x}_1 - x_1| + |\tilde{x}_2 - x_2|}$$

$$= \limsup_{\tilde{\mathbf{x}}-\mathbf{x}} \frac{|\tilde{x}_1 - x_1 + x_1 - x_2|}{|\tilde{x}_1 - x_1| + |\tilde{x}_2 - x_2|}$$

$$\leq \limsup_{\tilde{\mathbf{x}}-\mathbf{x}} \frac{|\tilde{x}_1 - x_1| + |x_1 - x_2|}{|\tilde{x}_1 - x_1| + |\tilde{x}_2 - x_2|}$$

$$= \limsup_{\tilde{\mathbf{x}}-\mathbf{x}} \left(\frac{1 + \left|\frac{x_1 - x_2}{\tilde{x}_1 - x_1}\right|}{1 + \left|\frac{\tilde{x}_2 - x_2}{\tilde{x}_1 - x_1}\right|} \right)$$

$$\leq 1 + \left|\frac{x_1 - x_2}{\tilde{x}_1 - x_1}\right| \to \infty$$

This potentially infinite error amplification for unfortunately disturbed input values is what makes the minimum function a mathematically ill-conditioned problem. This kind of behaviour is more famous for subtraction where disturbance of close input values can lead to a phenomenon known as catastrophic cancellation [11]. Having identified the ill-conditioned minimum function as the problematic part of the algorithm, a targeted mitigation strategy to improve the overall performance can be applied. Prior to the calculation of the Jaccard index, the instrument error is estimated as the mean standard deviation of the intensity values over

time. Therefore, before evaluating the minimum function, the difference of the two input values is compared against the estimated instrument error to find out if the evaluation will be negatively impacted by the disturbance. If the instrument error is smaller than the difference, the minimum function can be safely evaluated without further action. If the instrument error is greater though, the values will be equalised to avoid the error amplification from the minimum function. This is justifiable because the values are, indeed, the same within the error margin of the instrument. The exact implementation and the effect of the error correction mechanism on real experimental data can be found in section 6.1.

6.1 Effect of the error correction upon calculation of the Jaccard index

As described in mathematical detail in the main manuscript, the calculation of the Jaccard similarity index is very sensitive to experimental errors due to the bad conditioning of the minimum and maximum functions that are applied to obtain the intersection and union area of two spectra. When accounting for the experimental error correctly, the data can still be used for monitoring purposes though.

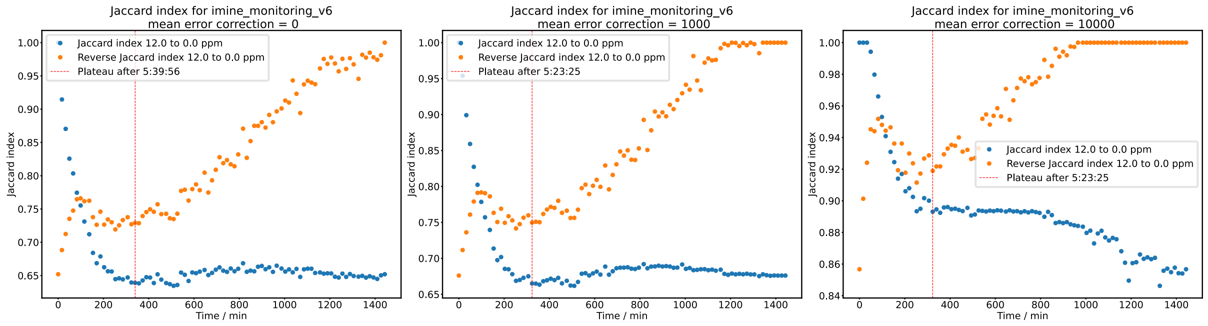

Figure S6: **Effect of the error correction on the calculation of the Jaccard index.** Taking the instrument error into account has a substantial effect on the evaluation of the ill-conditioned minimum function during the calculation of the Jaccard similarity index. The blue data series was obtained by calculating the Jaccard similarity index between the first and the current spectrum, the orange data series by comparing all spectra against the last spectrum. **From left to right**: no error correction, estimated mean error of 1×10^3, estimated mean error of 1×10^4.

Figure S6 illustrates how the error avoidance mechanism has a substantial effect on the regime where the compared spectra are very similar. The plot without error mitigation on the left-hand side starts fluctuating after some time especially when taking the last spectrum as a reference for the similarity index (orange data series). This effect is much less pronounced in the middle plot where the data was corrected against the mean standard deviation of the intensity values in the NMR spectra. When setting the threshold for error avoidance too high, there will be a loss of information though. Fortunately, the plateau detection, which is based on the data series that is using the first spectrum as a reference (blue series), is not greatly affected by the fluctuations coming from the error amplification in this specific case.

7 Comparison of different directionalities when calculating the similarity

In initial experiments, different options for choosing a reference point for the similarity index calculation were explored. When comparing the most recent spectra against each other (green data series in Figure S7), one would expect that the similarity index eventually approaches a value of 1.0 when the spectra do not change any more. The initial difference is too small for an efficient application of a slope detection algorithm though, which is why it is more practical to compare every spectrum against the first spectrum (blue series in Figure S7). Retrospectively, it is also possible to calculate the similarity between each spectrum and the last spectrum (orange series in Figure S7), but this is not a useful option for dynamic monitoring since the last spectrum is, naturally, unknown while conducting the experiment.

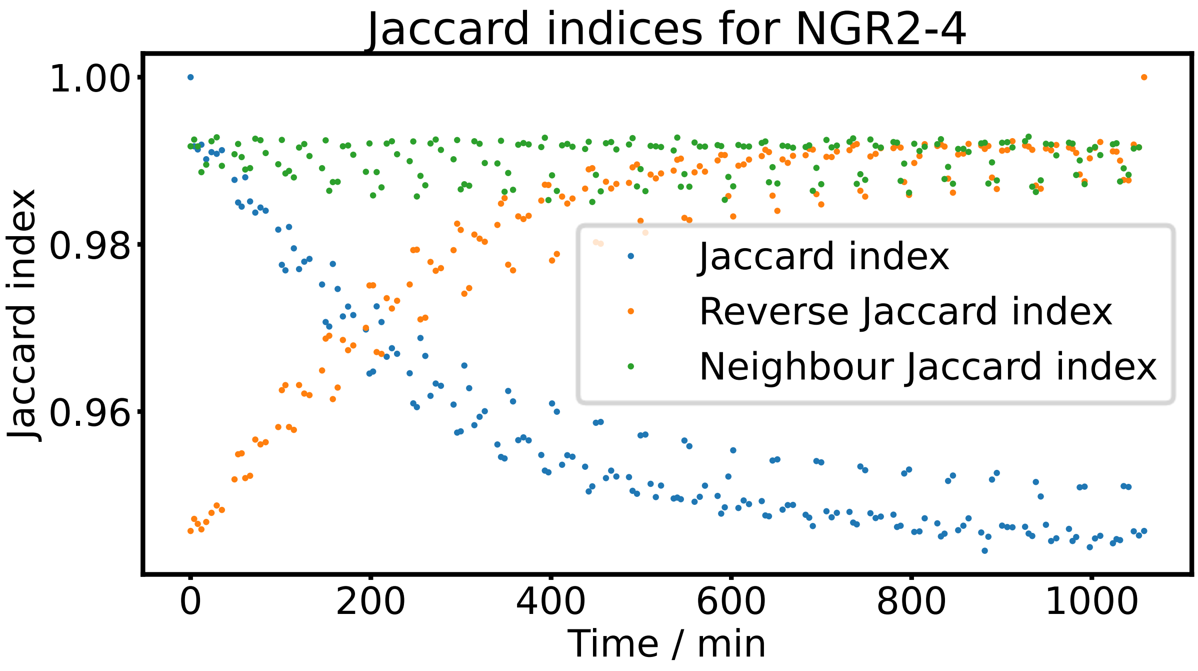

Figure S7: Different reference points for the similarity index led to different time series. It is not required to always reference the similarity index against the first spectrum in the data series. Experience has shown that using the first spectrum as a reference is the most practical option though.

8 Calculations for kinetic analysis

The formation of lophine was monitored at five different temperatures and a time series of the similarity index was plotted for each temperature.

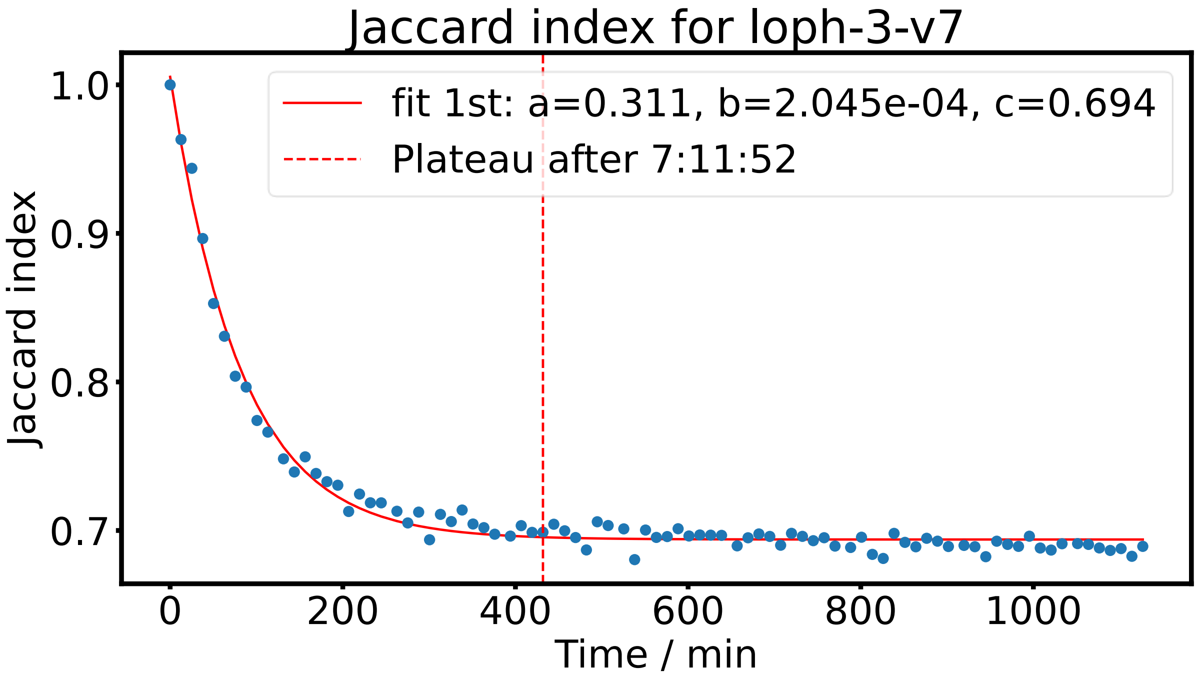

Figure S8: Kinetic analysis for the formation of lophine. The course of the similarity index follows an exponential decay function over time, from which a time constant can be derived that is indicative of the reaction rate.

To that plot, an exponential decay function $a \cdot \exp(-b \cdot x) + c$ was fitted and the decay constant b from that fit was, subsequently, plotted against the reaction temperature in an Arrhenius fashion [12,13] (Equation S4).

$$k = A \cdot \exp\left(-\frac{E_\mathrm{A}}{\mathrm{R}T}\right) \tag{S3}$$

$$\Leftrightarrow \ln k = -\frac{E_\mathrm{A}}{\mathrm{R}} \cdot \frac{1}{T} + \ln A \tag{S4}$$

9 References

1 Rohrbach, S. *et al.* Digitization and validation of a chemical synthesis literature database in the ChemPU. *Science* **377**, 172-180 (2022). https://doi.org:10.1126/science.abo0058

2 Head, T. *Async optimization Loop*, <https://scikit-optimize.github.io/0.8/auto examples/ask-and-tell.html> (2017).

3 Frazier, P. I. *A Tutorial on Bayesian Optimization*. (2018).

4 Garnett, R. *Bayesian Optimization*. (Cambridge University Press, 2023).

5 Matysiak, B. M., Thomas, D. & Cronin, L. Reaction Kinetics using a Chemputable Framework for Data Collection and Analysis. *Angewandte Chemie International Edition* **63**, e202315207 (2024). https://doi.org:https://doi.org/10.1002/anie.202315207

6 Pitre, S. P., McTiernan, C. D., Vine, W., DiPucchio, R., Grenier, M. & Scaiano, J. C. Visible-Light Actinometry and Intermittent Illumination as Convenient Tools to Study Ru(bpy)3Cl2 Mediated Photoredox Transformations. *Scientific Reports* **5**, 16397 (2015). https://doi.org:10.1038/srep16397

7 Adler, S. J. & Noyes, R. M. The Mechanism of the Permanganate-Oxalate Reaction. *Journal of the American Chemical Society* **77**, 2036-2042 (1955). https://doi.org:10.1021/ja01613a002

8 Randle, R. I., Cavalcanti, L., Sproules, S. & Draper, E. R. Aggregate dependent electrochromic properties of amino acid appended naphthalene diimides in water. *Materials Advances* **3**, 3326-3331 (2022). https://doi.org:10.1039/D2MA00207H

9 Rauschen, R., Ayme, J.-F., Matysiak, B. M., Thomas, D. & Cronin, L. A programmable modular robot for the synthesis of molecular machines. *Chem* https://doi.org:10.1016/j.chempr.2025.102504

10 Spearman, C. The Proof and Measurement of Association between Two Things. *The American Journal of Psychology* **15**, 72-101 (1904). https://doi.org:10.2307/1412159

11 Goldberg, D. What every computer scientist should know about floating-point arithmetic. *ACM Comput. Surv.* **23**, 5–48 (1991). https://doi.org:10.1145/103162.103163

12 Arrhenius, S. Über die Dissociationswärme und den Einfluss der Temperatur auf den Dissociationsgrad der Elektrolyte. **4U**, 96-116 (1889). https://doi.org:doi:10.1515/zpch-1889-0408

13 Arrhenius, S. Über die Reaktionsgeschwindigkeit bei der Inversion von Rohrzucker durch Säuren. **4U**, 226-248 (1889). https://doi.org:doi:10.1515/zpch-1889-0416
